# Revolutionizing drug delivery: low-intensity pulsed ultrasound (LIPUS)-driven deep penetration into hypoxic tumor microenvironments of cholangiocarcinoma

**DOI:** 10.7150/thno.99981

**Published:** 2025-01-01

**Authors:** Sera Hong, Jaihwan Kim, Gujin Chung, Donghyuk Lee, Joon Myong Song

**Affiliations:** 1 College of Pharmacy, Seoul National University, Seoul 08826, South Korea.; 2 Department of Internal Medicine, Seoul National University Bundang Hospital, Seoul National University College of Medicine, Seongnam 13620, South Korea.; 3 Seoul National University Bundang Hospital Healthcare Innovation Park 6F, Seongnam 13605, South Korea.

**Keywords:** Cancer imaging, LIPUS, Unidirectional fluid flow, Liposome, Chemotherapy

## Abstract

**Background:** Hypoxia is a major obstacle in the treatment of solid tumors because it causes immune escape and therapeutic resistance. Drug penetration into the hypoxic regions of tumor microenvironment (TME) is extremely limited. This study proposes using the unidirectional fluid flow property of low-intensity pulsed ultrasound (LIPUS) to overcome drug penetration limitations in the TME. LIPUS is gaining attention as a therapeutic modality for cancer owing to its safety and efficacy.

**Methods:** LIPUS parameters, such as the intensity, duty cycle (DC), and duration, were optimized to enhance drug delivery into the hypoxic regions of the TME in cholangiocarcinoma (CCA). Transparent tumor imaging using the tissue optical clearing method (CLARITY) enabled 3D visualization and quantitative assessment of drug delivery and therapeutic efficacy in relation to blood vessels in an intact tumor at the micrometer level. The antitumor efficacy of LIPUS-assisted chemotherapy was evaluated in a CCA xenograft mouse model.

**Results:** LIPUS significantly enhanced drug delivery efficacy into the hypoxic region of the TME in CCA. Under optimal conditions, i.e., a DC of 45% and a spatial-peak temporal-average intensity (Ispta) of 0.5 W/cm², drug penetration, including liposomal nanoparticles and chemotherapeutic agents gemcitabine and cisplatin, was improved by approximately 1.8-fold, resulting in a fivefold increase in apoptotic cancer cell death and a significant reduction in CCA growth. Notably, drug penetration and efficacy were more significantly affected by DC compared to the spatial-peak pulse-average intensity (Isppa). The efficacy saturated at Ispta values above 0.5 W/cm² under a 45% DC. Furthermore, we confirm that LIPUS induces non-thermal effects without causing cell damage, ensuring biosafety. These findings highlight the potential of LIPUS as a non-invasive strategy for treating hypoxic tumors.

**Conclusion:** LIPUS adjuvant therapy promises improved cancer treatment outcomes and offers a safe and innovative therapeutic strategy for CCA and other hypoxic tumors.

## Introduction

The tumor microenvironment (TME), where tumor cells reside, plays a significant role in tumor formation and progression within tumor tissue. The TME consists of tumor cells, tumor stromal cells (including cancer-associated fibroblasts (CAFs), tumor-associated macrophages (TAMs), and T or B lymphocytes), extracellular matrix (ECM), and secreted proteins 1. Tumor cells within the TME dynamically interact with stromal cells and the ECM, contributing to tumor evolution and the promotion of metastasis 1-4. The TME is characterized by irregular vascular networks, reduced blood flow, uneven perfusion, elevated interstitial fluid pressure (IFP), impaired lymphatic drainage, heterogeneous tumor vessel permeability, abundant stromal cells, and dense ECM and tumor cells 2, 5. These pathological TME conditions compromise clinical drug delivery to tumors, such as systemically administered chemotherapeutics and their carriers 6-8. Inadequate drug concentrations within tumors impede the effectiveness of anticancer drugs. Overcoming these challenges is crucial for effective cancer therapy.

Ultrasound therapy utilizes an inaudible range of mechanical pressure waves (> 20 kHz) and has a rich history of innovative applications in the medical field 9-17. Low-intensity pulsed ultrasound (LIPUS) is gaining attention as a therapeutic modality because it induces a relatively low thermal impact and fewer side effects on biological tissues than high-intensity and continuous energy 18. LIPUS stimulates the transport of anticancer drugs to the target cells or tissues 19, 20. One of the main mechanisms by which LIPUS enhances drug delivery efficacy is an increase in cell membrane permeability (sonoporation). Ultrasound-generated periodic pressure induces vibrations that temporarily open the cell membrane, thereby enhancing the uptake of anticancer drugs. The second mechanism involves the improvement of fluid flow within tissues. This is associated with the formation of acoustic streamlines resulting from pressure waves with a pattern of compression and rarefaction moving in a consistent direction within the fluid. This unidirectional fluid flow accelerates the transportation of drug particles to their intended destinations 2, 21.

Tumor hypoxia has emerged as a major issue in the diagnosis and treatment of cancer because of the obstacles in delivering chemotherapeutic drugs to these zones, which are characterized by decreased oxygen tension, aberrant vascular structure, and malignant gene expression 22-24. In such cases, external physical stimulation by ultrasound is effective for enhancing drug delivery to distant regions within the tumor. Recent studies have made notable progress in combining ultrasound with other strategies, such as oxygen-generating nanoparticles, microbubbles, and sonodynamic therapy, to enhance therapeutic efficacy in hypoxic tumor regions 11-14, 25-28. These approaches have demonstrated potential in temporarily alleviating hypoxia or improving therapeutic efficacy through mechanisms such as cavitation, reactive oxygen species production, or immunomodulation 10, 12. Despite these advancements, challenges persist, particularly in achieving enhanced drug penetration in poorly vascularized hypoxic regions. In this study, we employed the tissue optical clearing method CLARITY to evaluate the efficacy of drug delivery under LIPUS in a mouse model of cholangiocarcinoma (CCA). CLARITY, a technique for rendering tissues optically transparent, facilitates the spatial imaging analysis of intact biological structures without the need for tissue sectioning and reconstruction 29, 30. The presence of densely packed lipid bilayers in animal tissues gives rise to diffusion barriers and light scattering, thus hampering the penetration of light and macromolecules into deeper tissue layers 29, 30. However, CLARITY allows tissue transparency following the nondestructive lipid removal process, thereby enabling the acquisition of high-resolution three-dimensional (3D) imaging information from unsectioned tissues and providing insights into the distribution of drugs and hypoxic regions with respect to blood vessels within tumors 29. Therefore, the aim of this study was to evaluate the improvement in drug delivery efficacy by LIPUS in the hypoxic TME in a mouse model of CCA using transparent tumor imaging and to optimize the LIPUS parameters (intensity, duty cycle, duration, and power, among others) to provide an effective and economical cancer therapy technique compared to conventional chemotherapy or drug delivery systems (DDS). CCA begins in the bile duct and is the second most common type of primary liver cancer 31. The prognosis for CCA is unfavorable owing to the high prevalence of advanced-stage disease, with 75% of patients succumbing within a year of diagnosis and a dismal five-year survival rate below 20% 32. Surgical resection provides the only chance for a long-term cure; however, CCA is commonly diagnosed at advanced stages, and many patients are dependent on palliative systemic therapy 33. Gemcitabine (Gem) combined with cisplatin (Cis) (GP) has served as the first-line therapy for advanced or unresectable CCA; however, this standard chemotherapy regimen provides only modest benefits 34. Although recent combination therapy with Gem/Cis (GP) and immune check inhibitors has shown better survival outcomes than Gem/Cis (GP) alone, there is still room for improvement in this dismal disease 35, 36. To the best of our knowledge, this study is the first to demonstrate the penetration depth and number of apoptotic cells caused by systemically administered anticancer drugs in the hypoxic TME of CCA according to the treatment time, duty cycle (DC), and spatial-peak temporal-average intensity (Ispta) of LIPUS through 3D transparent tumor imaging. It also shows the spatial distribution of LIPUS-assisted apoptotic cancer cells and liposomal nanoparticles (NPs), the most widely used drug carriers in clinical settings as a DDS, in relation to the cancer cell density, irregular vasculature, and hypoxic region in the TME. Subsequently, the improved antitumor efficacy of LIPUS-assisted combination chemotherapy was evaluated *in vivo* under optimal LIPUS conditions derived from transparent tissue imaging.

## Materials and methods

### Cell culture

The CCA cell line, HuCCT1, was purchased from RIKEN BRC Cell Bank (RCB1960; Tsukuba, Japan). The cell line was maintained in RPMI 1640 medium (11875093; Gibco, Grand Island, NY, USA) supplemented with 10% fetal bovine serum (16000044; Gibco), 1% penicillin-streptomycin (15140122; Gibco) at 37 °C in a humidified incubator with a 5% CO_2_.

### Xenograft mice model

All animal experiments were performed in accordance with a protocol approved by the Institutional Animal Care and Use Committee (IACUC) of Seoul National University. Seven-week-old female BALB/c nude mice (approximately 20 g each) were purchased from Orient Bio, Inc. (Seongnam, Republic of Korea). To initiate tumor xenograft, 4 × 10^6^ HuCCT1 cells mixed with 100 μL Geltrex (A14132-02; Gibco) were implanted subcutaneously into the right flank area of each mouse.

### Liposome preparation

Liposomes-encapsulated ruthenium II (Ru-Lip) was prepared as described previously. Briefly, DOPC/DOTAP/DSPE-PEG-2000/cholesterol (60:5:5:30) was dissolved in chloroform, and a solution of Ru in methanol was added to the mixture. Ru was incorporated into the liposomes using the thin-film hydration method. The solvent was gradually removed by rotary evaporation until a thin film formed. The lipid film was then hydrated with PBS, and the resulting liposome dispersion in PBS underwent brief sonication. Unilamellar vesicles were created by extruding the liposomes through polycarbonate membranes with pore sizes of 0.2 μm and 0.1 μm (Avanti Polar Lipids, USA). To remove free Ru, the dispersion was centrifuged at 14,000 × g for 30 min using an Amicon Ultra centrifugal filter. Ru-encapsulated liposomes were freshly prepared for each experiment. The liposomes were characterized using a dynamic light scattering spectrophotometer (Zetasizer, Malvern Panalytical, Malvern, UK). The fluorescence signal of Ru was employed to visualize drug distribution within the TME. Through fluorescence imaging, the presence of Ru within the TME was observed, indirectly validating the efficacy of liposomal drug delivery. Additionally, Ru complex can bind to polyacrylamide hydrogel matrices used in tissue clearing protocols, ensuring its detectability during the clearing process. This enables accurate imaging of the intratumoral drug distribution without loss.

### Ultrasound application

The xenograft mice were anesthetized using ketamine and xylazine, and then subject to ultrasound treatment for 1 h (Figure 3A). To ensure proper acoustic coupling, ultrasound gel (Ecosonic, Sanipia, Republic of Korea) was applied to their skin. Ultrasound was delivered using a transducer (DEEPSONBIO Co., Ltd, Republic of Korea) operating at a fundamental frequency of 250 kHz in a pulsed manner (100 ms pulse length, 1 Hz pulse frequency, at a DC of 5%, 22%, 45% and Ispta of 0.1 W/cm^2^, 0.3 W/cm^2^, 0.5 W/cm^2^, 0.7 W/cm^2^) (Table S1). The ultrasound transducer, with an outer diameter of 56 mm, was powered by a Neuclare (B-01) (DEEPSONBIO Co., Ltd, Republic of Korea). The transducer used in our setup had a radius of curvature of 168 mm and a (axial) focal size of 55 mm. To focus the ultrasound on the CCA tumor-bearing xenograft mice, the transducer was coupled to a hydrogel-filled cone. The purpose of the cone was to facilitate coupling rather than focusing. By attaching the cone to the transducer, the setup for CCA xenograft mouse models was effectively adapted. The hydrogel within the cone helped to minimize output loss and ensured the efficient transmission of ultrasound waves. The acoustic intensity from the transducer surface in relation to the input voltage magnitude was calibrated using a calibrated hydrophone (HNR500 series, Onda, Sunnyvale, CA, USA) positioned 5 mm away from the transducer surface. The acoustic intensity profile was then recorded 5 mm away from the transducer surface (Figure S4).

### Dye infiltration test

Melamine foams (PK Global, Germany) were cut into dimensions of 40 × 40 × 30 mm (width × height × thickness). Agarose powder (A9414, low gelling temperature, Sigma) was dissolved in phosphate buffered saline (PBS) at a concentration of 2.5% w/w at 85 °C. The agarose solution was poured into a mold measuring 35 × 35 × 5 mm (width × height × thickness) and allowed to gel. After gelling, the agar block was immersed in TbO dye solution (0.25 mg/mL; T3260, Sigma). The melamine foams were hydrated in PBS, transferred to a beaker, and the agar block soaked in TbO dye solution was placed on top of the foam. LIPUS was applied to the front surface of the foam block for 1 h at an Ispta of 0.5 W/cm² and a DC of 45%. After sonication, the foam blocks were cut in half along the sonication axis and imaged using a smartphone (Galaxy S24+ plus, Samsung, South Korea).

### Membrane permeability test

Approximately 0.3 × 10⁶ HuCCT1 cells were seeded in a 6-well plate. After 24 h, the cells were treated with a 10 µM Ru solution and then subjected to LIPUS at an Ispta of 0.5 W/cm² with a DC of 45% for 1 h. Following irradiation, the cells were washed with PBS. Membrane permeability was assessed using confocal microscopy and flow cytometry to capture Ru fluorescence in HuCCT1 cells. The Ru signal area was quantified using ImageJ software (National Institutes of Health, USA) for confocal images and FlowJo software for flow cytometry analysis.

### Thermal analysis

Although low-intensity ultrasound (Ispta < 0.7 W/cm²) was used, thermal imaging was performed during LIPUS irradiation to assess tissue heating. Tumor-bearing mice were placed on a temperature-controlled heating pad to maintain the body temperature. A thermal imaging camera (FLIR ONE^®^ Pro, Teledyne FLIR LLC, USA) was positioned in front of the mice to capture temperature readings before, during, and 1 h after LIPUS exposure. The transducer was placed over the tumor and irradiated for 1 h at an Ispta of 0.5 W/cm² with a 45% DC. The highest and lowest temperature points within the irradiated area were marked for analysis.

To measure the temperature variation during LIPUS irradiation, the beaker containing cell culture media was placed in a water bath at 37 °C and irradiated with LIPUS (Ispta of 0.7 W/cm², 45% DC) for 1 h. Temperature changes in the media were monitored using a thermometer during the irradiation.

### Cytotoxicity assay

To perform the MTT assay, approximately 0.3 × 10^6^ HuCCT1 cells were seeded in a 6-well plate and incubated at 37 °C in 5% CO_2_ for 24 h. Cells were irradiated with LIPUS at different conditions (DCs of 5%, 22%, and 45% at Ispta levels of 0.5 W/cm^2^ and 0.7 W/cm^2^) for 1 h. After 24 h, the cells were incubated with a thiazoyl blue tetrazolium bromide (MTT) solution for 3 h at 1.2 mM. The cell viability was assessed by measuring the absorbance at 570 nm of the formazan/DMSO solution in each well, using an M5 multi-mode microplate reader (Molecular Devices, USA).

### Tissue sampling for studying the effect of LIPUS on drug delivery efficacy in TME

Experiments to determine the distribution of liposomal NPs were initiated when the tumor volumes reached approximately 1000 mm^3^. Liposome-encapsulated Ru (Ru-Lip, 8 mg/kg) was intravenously administered to HuCCT1 xenografts. 24 h after the intravenous injection, animals were anesthetized and locally exposed to ultrasound irradiation in a pulsed manner as mentioned above. The tumors were excised 48 h later. Immediately before tumor excision, DyLight 649 labeled tomato lectin (Vector Laboratories; excitation/emission: 649/665 nm) was injected via the tail vein (5 mg/kg). After 10 min, the animals were euthanized by CO_2_ asphyxiation. Tumors were washed with 1×PBS and fixed in 4% paraformaldehyde (PFA) overnight at 4 ˚C. Untreated mice (without irradiation: Ru-Lip injected into HuCCT1 xenografts) were used as negative controls.

Gem and Cis will be intravenously administered into HuCCT1 xenograft in the following manner: Gem, 200 mg/kg diluted in 0.1 mL saline intravenous injection; Cis, 5 mg/kg diluted in 0.1 mL saline intravenous injection. The animals were anesthetized with ketamine and xylazine 20 min or 24 h after intravenous drug injection. Tumors were locally exposed to the ultrasound irradiation in a pulsed manner as mentioned above. The dose of Gem/Cis used in the mice (per group, n = 4 to 5) was determined by referring to the conversion of the dose used in clinical patients through human-animal dose conversion. The tumors were excised 48 h later. Immediately before tumor excision, DyLight 649 labeled tomato lectin (Vector Laboratories; excitation/emission: 649/665 nm) was injected via the tail vein (5 mg/kg). After 10 min, the animals were euthanized by CO_2_ asphyxiation. Tumors were washed with 1×PBS and fixed in 4% PFA overnight at 4 °C.

### Tissue clearing

The PFA-fixed tumors were sliced into 1 mm thick sections and soaked in X-CLARITY hydrogel solution (Logos Biosystems) to ensure even diffusion of hydrogel monomers throughout the tissue at 4 °C for 24 h. Subsequently, nitrogen gas was used for 3 min to remove oxygen from the sample container. Tumor tissues were then polymerized by incubating the sample container in a 37 °C water bath for 3 h. Subsequently, the tumor samples underwent an active clearing process using an X-CLARITY tissue-clearing system (Logos Biosystems) with a current of 1.0 A at 37 °C. This system was employed alongside electrophoretic tissue clearing solution 1.2 L (Logos Biosystems) to expedite the elimination of lipids from the tissues through solution circulation. The tissue-clearing solution was based on SDS with a pH of 8.5. After the clearing process, the cleared tumor tissues were rinsed with 1×PBS for 2 d at 37 °C.

### Immunostaining for apoptotic cell death detection in HuCCT1 CCA

To detect LIPUS-assisted apoptotic cancer cells, cleared tumor tissues of 1 mm thickness were stained using a TUNEL staining kit (Takara Bio, Shiga, Japan). First, the tumor tissues were incubated in a permeabilization buffer at 4 °C for 4 h. The tissues were then incubated overnight at 37 °C in a tabletop incubator with a staining solution containing TdT enzyme, Hoechst 33342, and labeling safe buffer, followed by a wash with 1× PBS at 37 °C. Finally, stained samples were rinsed in DDW three times and immersed in X-CLARITY™ mounting solution (refractive index = 1.46, Logos Biosystems) at RT before confocal imaging.

### Immunostaining for Hif-1α expression in HuCCT1 CCA

For the detection of Hif-1α-expressive cancer cells in TME, cleared tumor tissues of 1 mm thickness were stained using Hif-1α monoclonal antibody in labeling solution (6% (vol/vol) BSA, 0.2% (vol/vol) Triton X-100, 0.01% (vol/vol) sodium azide, 1×PBS), followed by incubation with washing with 0.2% PBST. The samples were stained with Hoechst 33342 solution and washed with 0.2% PBST in a shaking chamber. The stained samples were rinsed in DDW three times and placed in X-CLARITY™ mounting solution (RI = 1.46, Logos Biosystems) at RT before confocal imaging.

### 3D imaging for distribution of Ru-Lip, Hif-1α, and apoptotic cell death in HuCCT1 CCA

The cleared tumor tissue, after incubation in the mounting solution, was positioned between a cover glass and the bottom of confocal dishes. Imaging of the cleared tumor tissues was performed using a Leica TCS SP8 DMI8-CS system equipped with an HC PL APO CS 10×/0.40 DRY objective (Leica Microsystems GmbH, Wetzlar, Germany). 3D fluorescence images were acquired using the system's Z-stacking function (Hoechst 33342: 405 nm, Ru-Lip/TUNEL: 488 nm, Hif-1α: 594 nm, and blood vessel: 633 nm excitation). The X/Y resolution was set at 1024 × 1024 pixels, with a scan depth of approximately 350 µm.

### 3D image analysis for distribution of Ru-Lip in HuCCT1 CCA

The 3D fluorescence images were analyzed using Imaris CL and XT software (Bitplane AG, Zürich, Switzerland) to assess the distribution of Ru-Lip in relation to the blood vessels. In the software, the 'spots' tool was initially utilized to count nuclei. For this, channel 1 (blue) was chosen to isolate a specific region of interest (ROI) in the algorithm step of the 'spots' application. The estimated diameter for spot detection was set to 5 μm, determined using the measurement point function. Next, the quality filter value was manually adjusted to be greater than 10 in the lower-threshold control box. Then, the 'surface' tool was employed for blood vessel segmentation. The ROI encompassed the entire image, and channel 3 (blood vessel, red) was the source channel. Blood vessels were segmented by setting a threshold after applying an optimal smoothing filter. Subsequently, the 'surface' tool was used to segment the membranes of Ru-Lip-bound cancer cells. Once again, the ROI was set to encompass the entire image, and channel 2 (Ru-Lip: green channel) was chosen as the source channel. The Ru-Lip signal was segmented by manually setting a threshold, followed by smoothing with an optimal filter. Finally, nuclei surrounded by Ru-Lip were identified through the 'find spots close to surface' tool, and a 3D distance transformation was computationally conducted for spots near the Ru-Lip surface outside the segmented vessels. The penetration of Ru-Lip from the intratumoral blood vessels was measured at the micrometer level in the TME. Based on the 3D-reconstructed images under all conditions, graphical data were generated to show the number of cancer cells that took up Ru-Lip in relation to their distance from the blood vessel.

### 3D image analysis for Hif-1α expression and apoptotic cell death in HuCCT1 CCA

To assess the distribution of apoptotic cell death in relation to the blood vessels, a 3D-reconstructed image was created using the IMARIS software. As mentioned previously, spot and surface applications were used to determine the distribution of apoptotic cells in the TME. In brief, first, nuclei were quantitatively determined using the 'spots' tool. For this, channel 1 (nuclei signal, blue) was chosen for the 'segment only a region of interest (ROI)' function during the image processing step of the spot tool, covering the entire image. The 'estimated diameter' for spot detection was set to 5 μm. Then, the quality filter value was manually adjusted to be greater than 5 in the lower threshold control box. Second, the 'surface' tool was utilized for blood vessel segmentation. The ROI encompassed the entire image, and the source channel was selected for channel 4 (red signal). The blood vessel signal was segmented by manually setting a threshold (background subtraction) after applying an optimal smoothing filter. Third, the 'surface' tool was applied to determine the number of nuclei with apoptotic signal (TUNEL) or Hif-1α positivity (channel 2 - green or channel 3 - magenta). The ROI was set to encompass the entire image, and channel 2 or 3 was chosen as the source channel. The apoptotic or Hif-1α signal was segmented by manually setting a threshold (background subtraction), followed by smoothing with an optimal filter. Lastly, nuclei enclosed by apoptotic or Hif-1α spots can be identified via the 'find spots close to surface' tool, and 3D distance transformation was computationally conducted outside the segmented vessels with spots close to apoptotic or Hif-1α nuclei. 3D distribution of apoptotic cell death or Hif-1α-expressing cancer cells was measured from the blood vessel at the micrometer level in TME. Analysis was performed on three different sets of tumor tissues, and the average values were presented.

Finally, to identify the distribution of apoptotic Hif-1α-positive cancer cells in relation to blood vessels, apoptotic nuclei spots co-localized with Hif-1α-positive spots were selected. The distance distribution of these apoptotic Hif-1α-positive cancer cells from blood vessels was then automatically calculated using the software.

### *In vivo* efficacy study

Experiments to determine the antitumor efficacy (tumor growth inhibition) of ultrasound in Gem and Cis treatments were initiated when the tumor volume reached 150 - 200 mm^3^. Tumor volume was measured using calipers every other day and calculated using the formula.

V = (W^2^ × L)/2

Where W and L represent the width and length of the tumor. When tumors reached a volume of 150 - 200 mm^3^, the mice were randomized into five groups of 25 mice: group 1, control; group 2, Gem plus Cis; group 3, Gem plus Cis with ultrasound therapy (0.5 W/cm^2^ Ispta, 22% DC); group 4, Gem plus Cis with ultrasound therapy (0.7 W/cm^2^ Ispta, 45% DC); group 5, Gem plus Cis with ultrasound therapy (0.5 W/cm^2^ Ispta, 45% DC). Cis (5 mg/kg) and Gem (200 mg/kg) were intravenously injected into the mice on days 1 and 8 for 21 days. Doses were determined and modified based on pharmaceutical company recommendations, with reference to the FDA-guided conversion equation of the human dose to the animal dose based on the body surface area 37. Ultrasonography was performed on days 2 and 9, 24 h after drug treatment. The tumor size was measured once every other day and compared among the five groups.

### Blood biochemistry test

We measured the serum levels of alanine transaminase (ALT) and aspartate transaminase (AST) for liver function, blood urea nitrogen (BUN) and creatinine (CRE) for kidney function, and creatine phosphokinase (CPK) and lactate dehydrogenase (LDH) for muscle injury and tissue damage, respectively. Blood samples from control and LIPUS-treated mice were centrifuged at 3,000 rpm for 10 min, and the serum was analyzed using a biochemical analyzer (Dri-chem 3500s, Fujifilm, Japan).

### Wound healing assay

HuCCT1 cells were seeded in confocal dishes and scratched with a pipette tip to create a wound. Cells were then treated with or without LIPUS (0.5 W/cm² Ispta, 45% DC). Wound closure was monitored by bright-field imaging at 0, 6, and 12 h. Cell migration was quantified by measuring the reduction in the wound area using ImageJ software (National Institutes of Health, USA).

### Statistical analysis

The data were analyzed by GraphPad Prism 9 (GraphPad Software Inc., La Jolla, CA, USA) using a one-way ANOVA with Tukey's post-hoc test or Student's t-test. Results were expressed as the mean ± SD (n = 3) and considered significant if P < .05.

## Results

### Evaluation of mechanical and thermal effects of LIPUS

**Figure 2:**
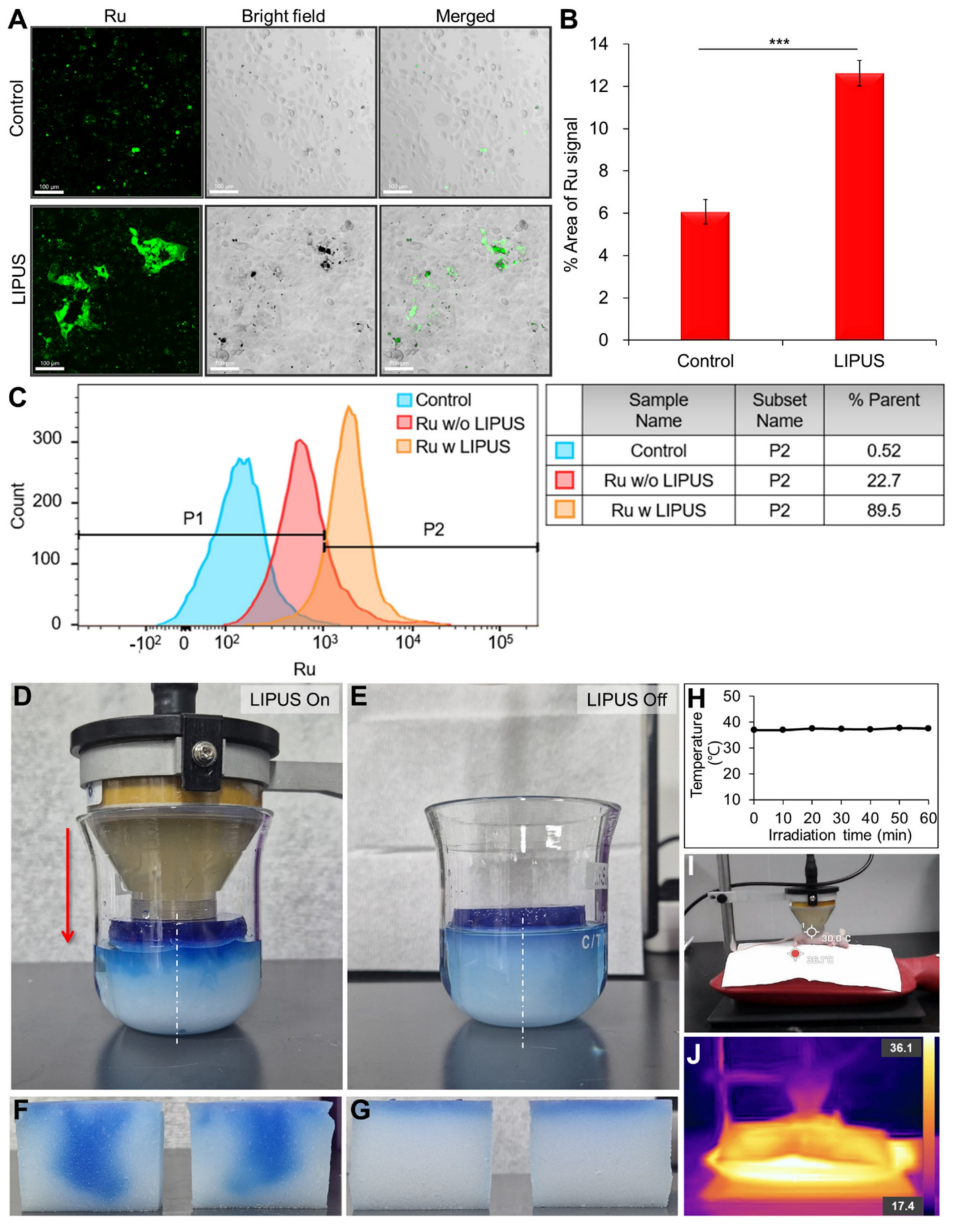
Mechanisms of LIPUS in enhancing drug penetration. (A-C) Cell membrane permeability (sonoporation) test. (A) Detection of the permeability of HuCCT1 cell membranes immediately after LIPUS irradiation with ruthenium (Ru) using confocal microscopy. (B) Quantification of the Ru signal area from the acquired cell images, presented as mean value ± SD (n = 3). Scale bar, 100 μm. *** P < 0.001. (C) Flow cytometric analysis of Ru in HuCCT1 cells: control, Ru treatment, and Ru treatment with LIPUS irradiation. (D-G) Unidirectional fluid flow property - examples of dye infiltration. (D) Experimental setup (front view) with a hydrogel-filled cone-coupled ultrasound transducer. (E) Experimental setup (front view) without an ultrasound transducer. (F, G) Dye infiltration depth in the middle sections of melamine foam (dashed line from the front view) after (F) LIPUS irradiation and (G) no LIPUS exposure. (H-J) Thermal effect tests. (H) Temperature variation in the cell culture medium during LIPUS irradiation (at 45% DC and 0.7 W/cm^2^ Ispta), as assessed by a thermometer. (I) Real image and (J) thermal image of a CCA tumor-bearing mouse 1 h after LIPUS irradiation. Temperature points marked with red dots and blank dots represent the temperatures on the heating pad (the highest temperature point) and in the mouse xenograft tumor, respectively.

Sonoporation was investigated by comparing Ru uptake in HuCCT1 cells subjected to LIPUS treatment. The analysis revealed a significant increase in cell membrane permeability (P < 0.001, Figure 2A-B), corroborated by flow cytometry, which indicated a notable enhancement in Ru signal in the LIPUS-treated group (Figure 2C). Figure 2B shows that the signal area of Ru has increased by more than two-fold compared to the control group. As shown in Figure 2C, the population of Ru-uptake HuCCT1 cells increased from 22.7% to 89.5% after ultrasound irradiation. Unidirectional flow properties induced by LIPUS were further assessed through dye infiltration experiments (Figure 2D-G) to examine the potential of acoustic streaming to enhance fluid flow into porous materials using melamine foam and agar hydrogel blocks. TbO dye infiltration was evaluated under the LIPUS condition with an Ispta of 0.5 W/cm^2^ and a DC of 45%. As a result, significant dye infiltration was observed in LIPUS-irradiated agar hydrogel blocks, indicating the potential of LIPUS to enhance fluid flow within the TME. Furthermore, we confirm that no significant heat generation occurred during LIPUS exposure both *in vitro* (Figure 2H) and *in vivo* (Figure 2I-J). Thermal imaging of CCA tumor-bearing mice showed no notable temperature increase at the tumor site after LIPUS irradiation.

### Evaluation of LIPUS-mediated anti-cancer drug delivery and efficacy in various DCs

**Figure 3:**
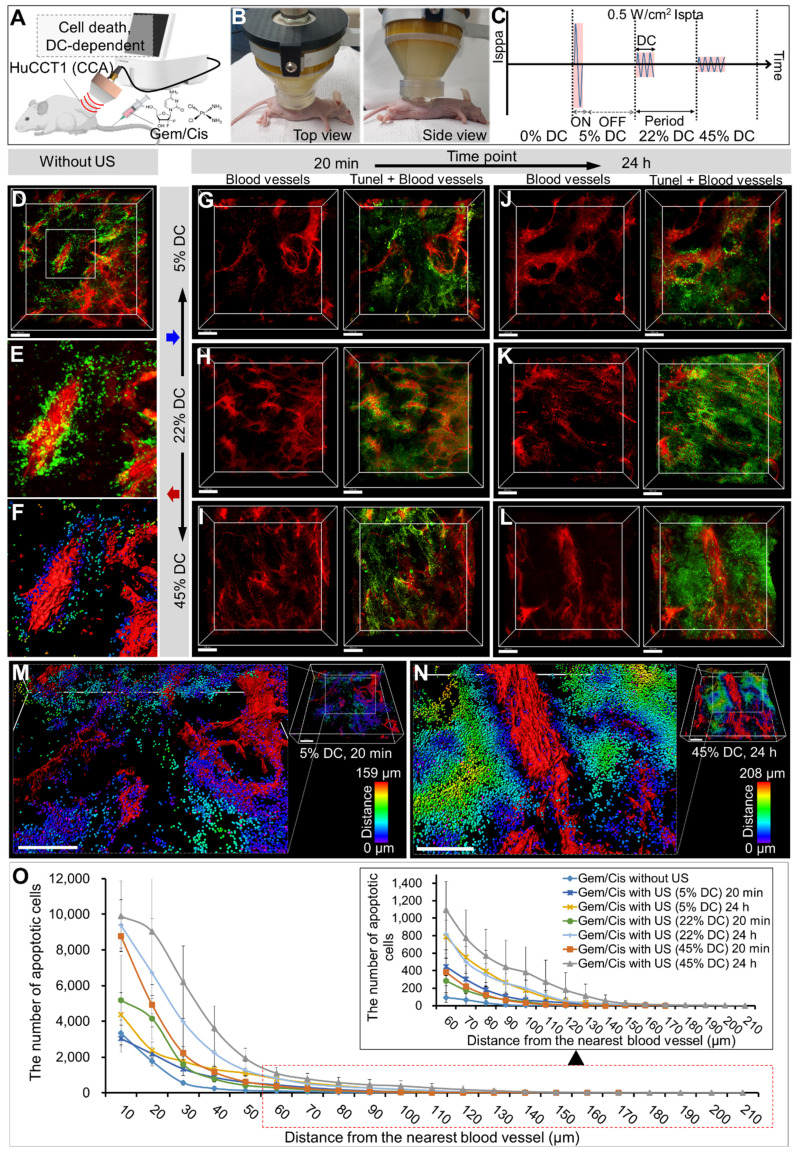
3D transparent tumor imaging showing the abnormal vascular network and spatial distribution of LIPUS-mediated anticancer drugs-induced apoptotic cancer cell death in CCA. A schematic diagram illustrating (A) LIPUS treatment in Gem/Cis-treated HuCCT1 tumor-bearing mice, including (B) a photo of a mouse receiving LIPUS and (C) variations in DC and spatial-peak pulse-average intensity (Isppa) under the identical Ispta. (D) 3D blood vessel image (red) with terminal deoxynucleotidyl transferase dUTP nick-end labelling (TUNEL) staining (green), including (E) a magnified view and (F) reconstructed image, acquired from Gem/Cis-treated HuCCT1 tumor-bearing mice (control) without LIPUS irradiation. (G-L) 3D blood vessel images (red) with TUNEL staining (green) acquired from mice receiving LIPUS at 5%, 22%, and 45% DCs either 20 min or 24 h post-drug injection. (G) 5% DC of LIPUS 20 min after drug injection. (H) 22% DC of LIPUS 20 min after drug injection. (I) 45% DC of LIPUS 20 min after drug injection. (J) 5% DC of LIPUS 24 h after drug injection. (K) 22% DC of LIPUS 24 h after drug injection. (L) 45% DC of LIPUS 24 h after drug injection. (M, N) 3D images of 3G and 3L were reconstructed, and apoptotic cell death within the TME was quantitatively analyzed using IMARIS software. (M) and (N) display the 3D-reconstructed images of 3G and 3L (raw images), respectively. Spots (apoptotic cancer cells) are depicted in spectral colors based on their proximity to the nearest blood vessel. All scale bars, 200 μm. (O) Graphical data showing the number of apoptotic cells in relation to the distance from the blood vessel between different DC conditions.

**Figure 4:**
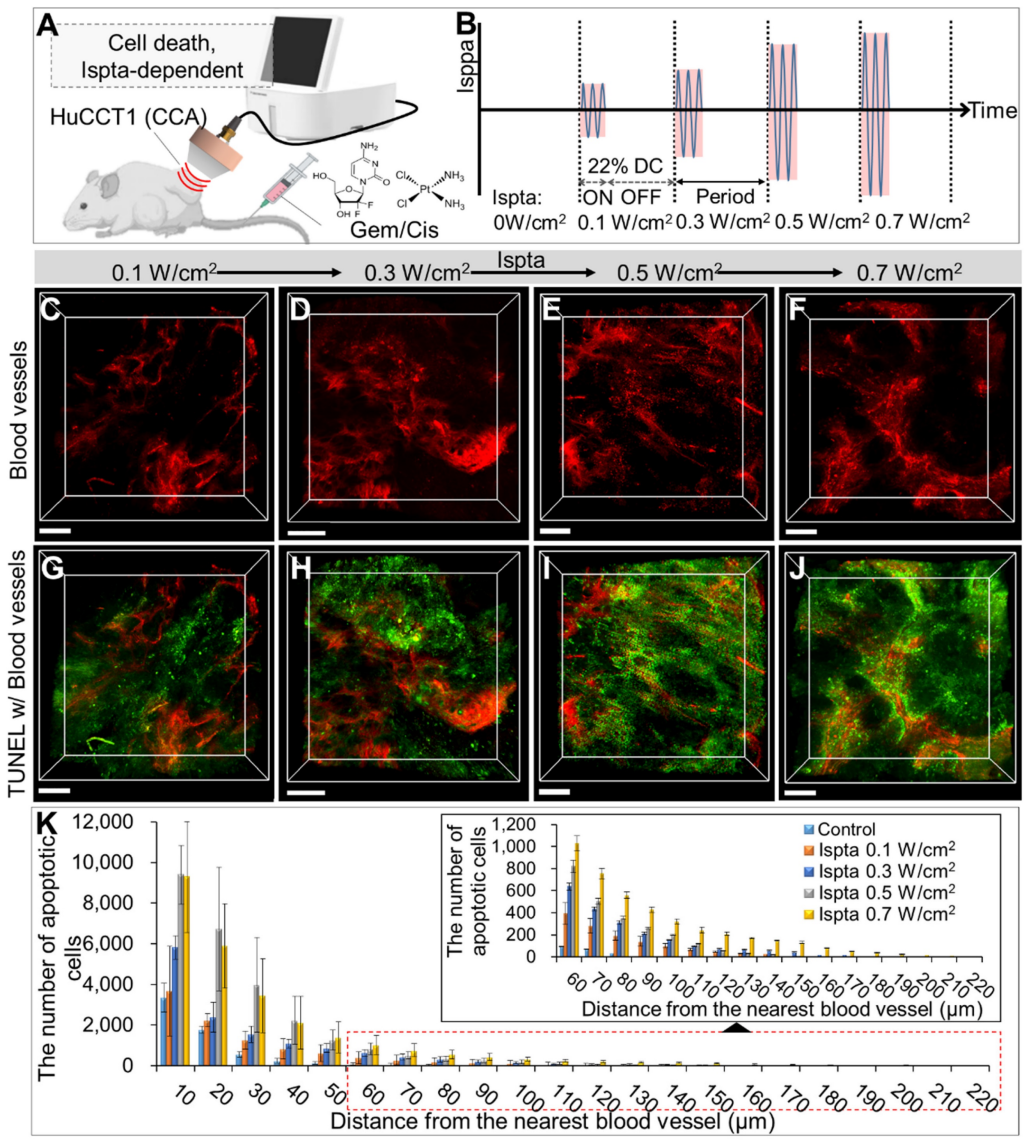
3D transparent tumor images from CCA-bearing mice receiving LIPUS at 0.1, 0.3, 0.5, and 0.7 W/cm^2^ of Ispta 24 h post-anticancer drug injection. A schematic diagram illustrating (A) LIPUS treatment in Gem/Cis-treated HuCCT1 tumor-bearing mice under (B) variations in Ispta. (C-F) 3D blood vessel images (red) with TUNEL staining (green) acquired from mice receiving LIPUS at (C, G) 0.1, (D, H) 0.3, (E, I) 0.5 and (F, J) 0.7 W/cm^2^ of Ispta. All scale bars, 200 μm. (K) Graphical data showing the number of apoptotic cells in relation to the distance from the blood vessel between different Ispta conditions.

The 3D images from CLARITY revealed an aberrant vascular network and distribution of LIPUS-mediated drug-induced apoptotic cancer cells in the CCA (HuCCT1 mouse xenograft tumor) (Figures 3 and 4). Based on the co-localization of blood vessels and TUNEL signals, apoptotic cancer cells were primarily observed in the perivascular region of the control group (Figure 3D-F). Comparing groups treated with LIPUS under varying DCs of 5%, 22%, and 45% at a constant Ispta of 0.5 W/cm^2^ (Figure 3C), an increase in DC correlated with an increase in Gem/Cis-induced apoptotic cell death (Figure 3G-L and Figure S1). An Ispta value of 0.5 W/cm² was chosen as it aligns with FDA safety standards that limit Ispta to 0.72 W/cm² for diagnostic ultrasound. This value ensures safety while maintaining clinical relevance and effectiveness 38-40. The apoptotic cell death of LIPUS-mediated chemotherapy increased more dramatically when administered 24 h after drug injection (Figure 3G vs. 3J, 3H vs. 3K, and 3I vs. 3L), compared to LIPUS treatment at 20 min after drug injection. Severe apoptotic cell death was observed in a 45% DC (24 h) (Figure 3L). Figure 3M-N shows representative 3D-reconstructed images demonstrating the penetration gradient of the apoptotic cancer cells in relation to blood vessels under 5% DC at 20 min and 45% DC at 24 h, respectively. The number of spots and maximum penetration distance were much higher in the 45% DC (24 h post-injection) compared to the DC of 5% (20 min post-injection) (32458 vs. 11904, 208 μm vs. 159 μm). Figure 3O and Table S3 compare the apoptotic cancer cell distribution under different ultrasound conditions. At equivalent distances from the blood vessels, the larger the DC, the greater the number of apoptotic cancer cells, and cancer cell death spreads further from the blood vessels. Tumor tissues treated with ultrasound alone, without anticancer drugs, did not exhibit significant cell necrosis under the specified LIPUS conditions (Figure S2).

### Evaluation of LIPUS-mediated anti-cancer drug delivery and efficacy in various Ispta levels

Comparing LIPUS-treated groups at Ispta of 0.1, 0.3, 0.5, and 0.7 W/cm^2^, with a DC of 22% (Figure 4A), showed higher Ispta correlated with increased drug-induced apoptotic cell death (Figure 4C-J). The DC of 22% was chosen based on previous findings demonstrating its efficacy in the non-invasive disruption of plasma protein binding (at 250 kHz LIPUS) and its common use in LIPUS studies 41-43, where DCs typically range from 5 to 50%, allowing for effective physiological responses without excessive thermal damage 44. Quantitative analysis revealed more apoptotic cells in the LIPUS-treated groups with higher Ispta values than in the control group at equivalent distances from the blood vessels, further extending the maximum distance observed (Figure 4K and Table S4). The Ispta 0.7 W/cm^2^ condition resulted in greater drug dispersion than 0.5 W/cm^2^, especially in regions distant from blood vessels (> 50 μm), though the overall quantity of apoptotic cancer cells remained similar (Table S4).

### Evaluation of LIPUS-mediated liposome NPs delivery in various DCs

**Figure 5:**
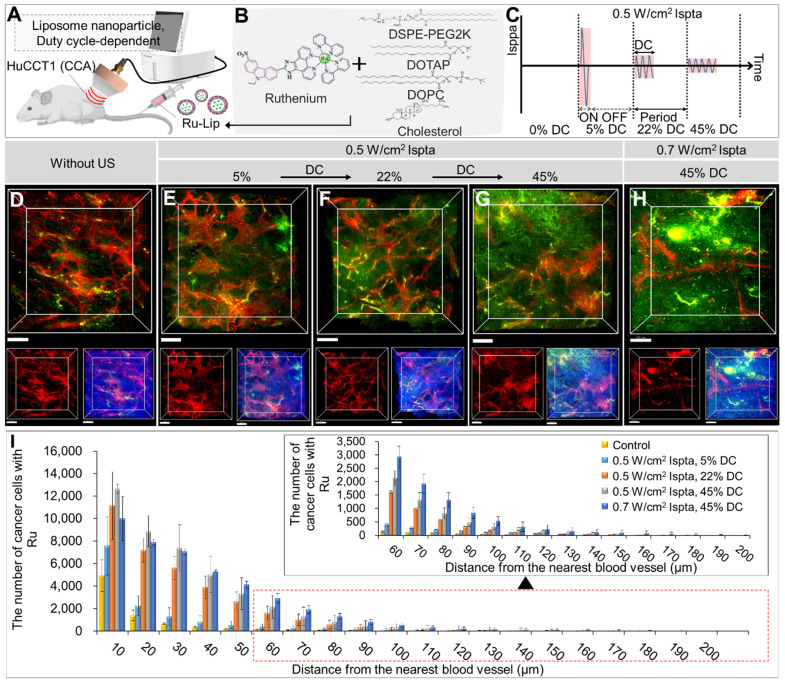
3D transparent tumor images from CCA-bearing mice receiving LIPUS at 5%, 22%, and 45% of DCs 24 h post-liposomal nanoparticle (Ru-Lip) injection. A schematic diagram illustrating (A, B) LIPUS treatment in Ru-Lip-treated HuCCT1 tumor-bearing mice under (C) variations in DC and Ispta. (D-H) 3D blood vessel images (red) with Ru-Lip (green) acquired from mice receiving LIPUS at (D) 0%, (E) 5%, (F) 22%, (G) 45%, and (H) 45% of DCs (D-G: 0.5 W/cm^2^ Ispta, H: 0.7 W/cm^2^ Ispta). (Top) 3D distribution of blood vessel (red) and Ru-Lip (green), (bottom left) blood vessels alone (red), and (bottom right) blood vessels (red) with Ru-Lip (green) and nucleus (blue). All scale bars, 200 μm. (I) Graphical data showing the number of cancer cells taken up by Ru-Lip in relation to the distance from the blood vessel among different DC conditions.

Figure 5 and Table S5 show *in vivo* liposomal NPs distributions under different DCs (5%, 22%, 45%) at 0.5 W/cm^2^ Ispta. Ru-Lip was characterized by encapsulation efficacy, particle size, and zeta potential (Figure S3 and Table S2). Ru-Lip distribution in the TME was visualized using a fluorescent Ru complex (Figure 5A-H). Higher DC application led to an increased Ru distribution (Figure 5D-H). Quantitative analysis showed that elevated cancer cells took up Ru at equivalent distances from blood vessels with higher DCs (Figure 5I and Table S5). At 0.7 W/cm^2^ Ispta compared to 0.5 W/cm^2^ Ispta (at DC 45%), there was an increased number of cancer cells taking up Ru in regions distant from blood vessels (> 50 μm). However, when Ispta exceeded 0.5 W/cm^2^, total cancer cell uptake of Ru became saturated.

### Evaluation of LIPUS-mediated liposome NPs delivery in various Ispta levels

**Figure 6:**
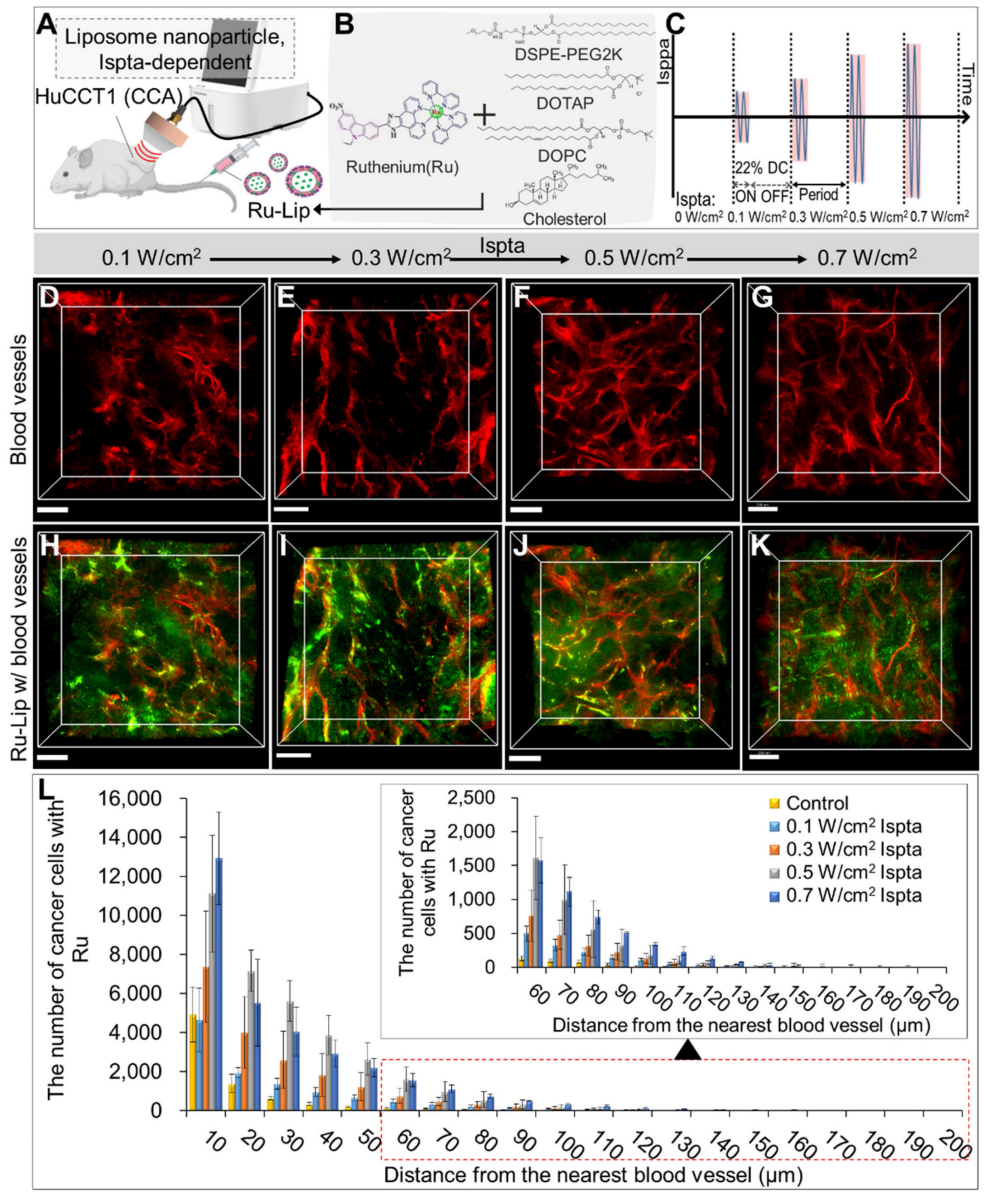
3D transparent tumor images from CCA-bearing mice receiving LIPUS at 0.1, 0.3, 0.5, and 0.7 W/cm^2^ of Ispta 24 h post-liposomal nanoparticle (Ru-Lip) injection. A schematic diagram illustrating (A, B) LIPUS treatment in Ru-Lip-treated HuCCT1 tumor-bearing mice under (C) variations in Ispta. (D-K) 3D blood vessel images (red) with Ru-Lip (green) acquired from mice receiving LIPUS at (D, H) 0.1, (E, I) 0.3, (F, J) 0.5, (G, K) 0.7 W/cm^2^ of Ispta. All scale bars, 200 μm. (L). Graphical data showing the number of cancer cells taken up Ru-Lip in relation to the distance from the blood vessel among different Ispta conditions.

Figure 6 illustrates LIPUS-mediated Ru-Lip NPs distribution at a DC of 22% with varied Ispta (0.1, 0.3, 0.5, 0.7 W/cm^2^) (Figure 6A-C). In the untreated control group, the Ru-Lip NPs mainly remained near the blood vessels (Figure 5D); however, with increasing Ispta levels, there was more diffusion beyond the vessels (Figure 6D-K). Higher Ispta levels resulted in an increased distribution of Ru-absorbing cancer cells at equivalent distances from the vessels (Figure 6L and Table S6). However, the Ru distribution in the TME reached saturation above 0.5 W/cm^2^ Ispta.

### LIPUS-mediated anti-cancer drug delivery and efficacy evaluation deep into the hypoxic region in TME of CCA

**Figure 7:**
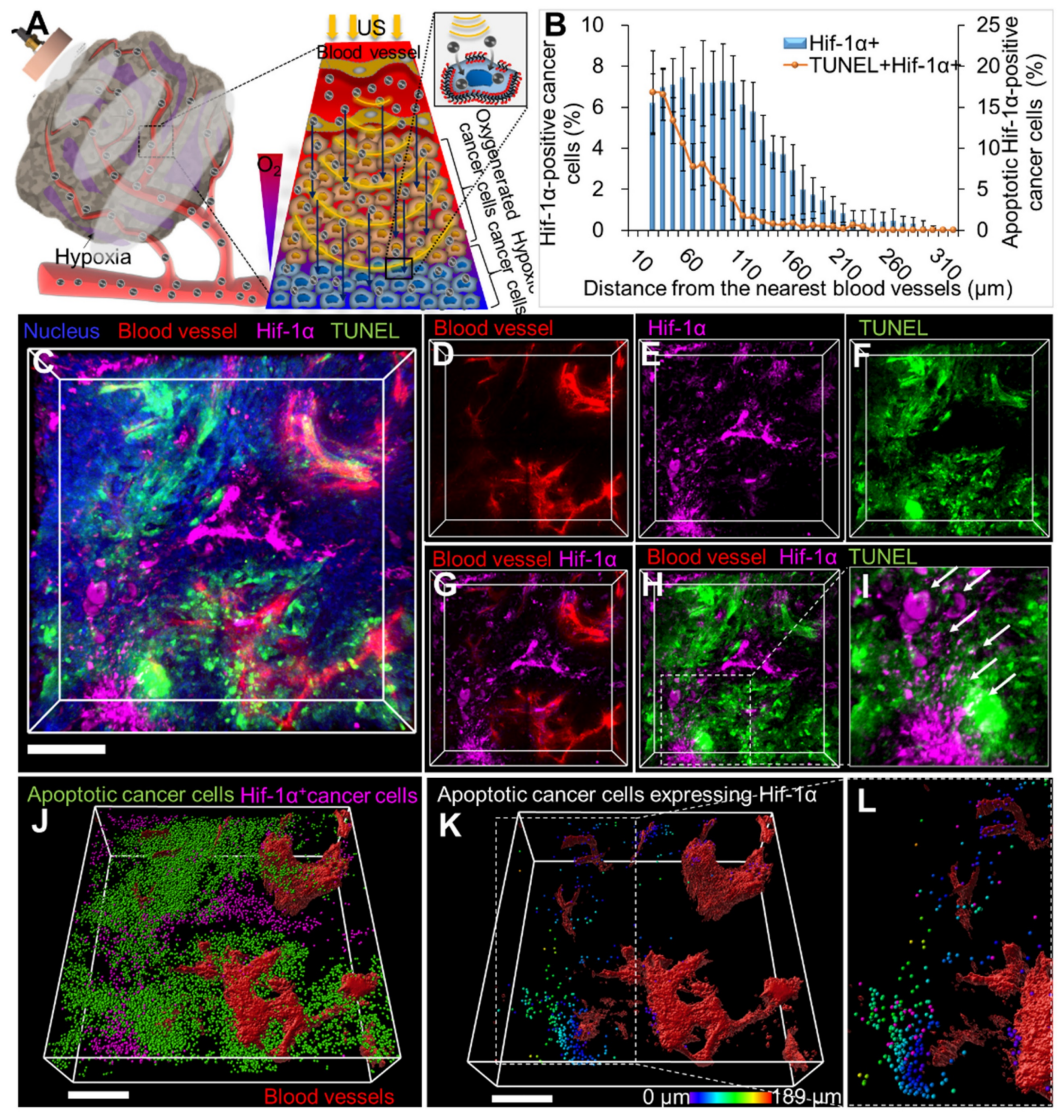
Spatial distribution of hypoxic cancer cells and apoptotic cancer cells in hypoxic regions of CCA following tissue clearing. (A) Schematic diagram showing the movement of drugs into hypoxic areas by unidirectional fluid flow and sonoporation of LIPUS (at 0.5 W/cm^2^ Ispta and 45% DC). (B) Graphical data signifies the percentage of Hif-1α-expressing cells and apoptotic Hif-1α-positive cells in relation to their distance from the blood vessel. (C) Tumor tissues were cleared and immunostained for Hif-1α cells (magenta), blood vessels (red), apoptosis (TUNEL, green), and nucleus (blue). (D) Blood vessels (red). (E) Hif-1α cells (magenta). (F) Apoptotic cancer cell death (green). (G) Both Hif-1α cells and blood vessels. (H) Blood vessels along with Hif-1α cells and apoptotic cells. (I) Magnified image of H (white arrow indicates apoptotic cells expressing Hif-1α). (J) 3D reconstructed image of Hif-1α-expressing cells (magenta spots) and apoptotic cancer cells (green spots) with 3D-vessel structure (red). (K) Apoptotic cancer cells (green) co-localized with Hif-1α expressing cells (magenta) designated by spectral spots relative to blood vessels. As distance increases, color shifts from blue to red. (L) Magnified image of K. All scale bars, 200 μm.

LIPUS-mediated delivery of Gem/Cis into hypoxic tumors was visualized via 3D transparent imaging (Figure 7). Hif-1α expression was observed distally from blood vessels, with apoptotic cell death (TUNEL) in extravascular areas (Figure 7D-G). Colocalization between the TUNEL signal and Hif-1α expression confirmed drug delivery deep into hypoxic cancer cells (Figure 7H-I). Hif-1α-positive cancer cells were found approximately 30-330 μm away from blood vessels (Figure 7B). The maximum distance from blood vessels to Hif-1α-positive apoptotic cancer cells was about 190 μm (Figure 7K, magnified Figure 7L).

### Comparison of the distribution of drug-induced apoptotic cancer cell death and liposomal NPs under varying DC and Ispta levels

**Figure 8:**
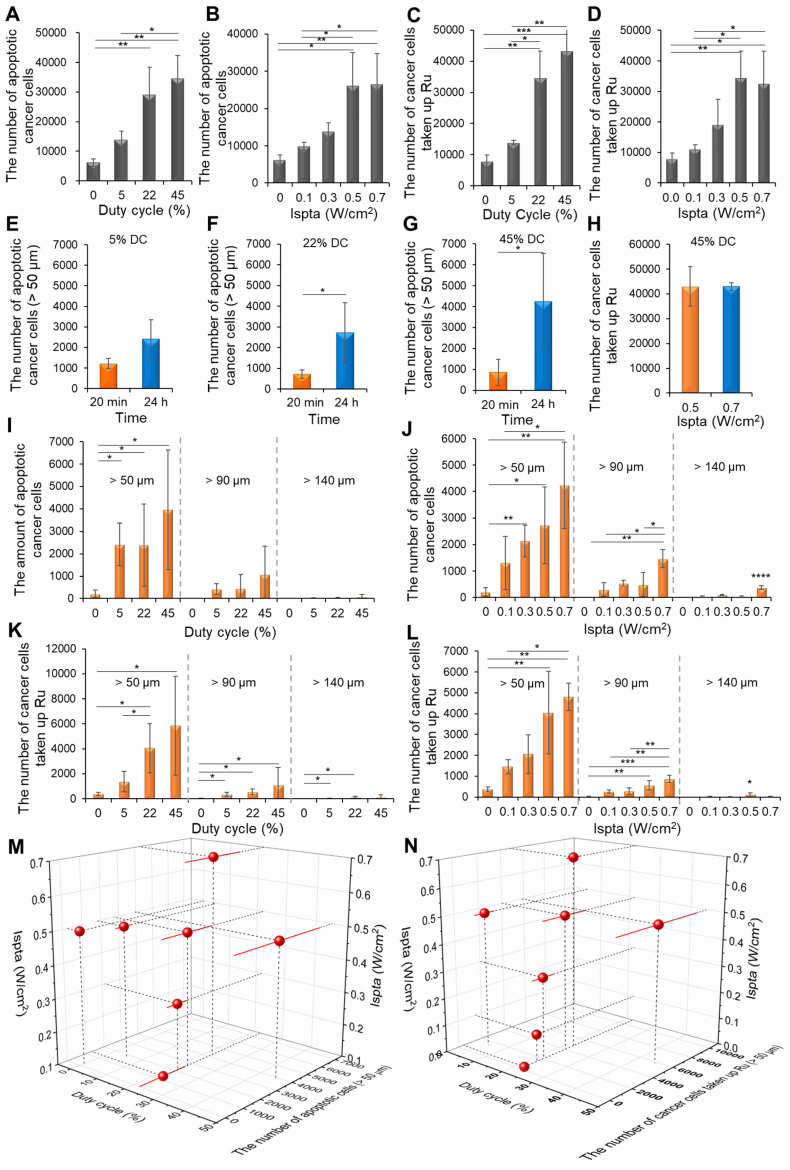
Statistical analysis of LIPUS-mediated Gem/Cis-induced cell death and Ru-Lip uptake by cancer cells at different DCs and Ispta levels. (A) Following Gem/Cis administration, with LIPUS applied 24 h later, the number of apoptotic cancer cells at various DCs under 0.5 W/cm² Ispta was analyzed. (B) Following Gem/Cis administration, with LIPUS applied 24 h later, the number of apoptotic cancer cells at different Ispta levels under 22% DC was analyzed. (C) The uptake of Ru-Lip by total cancer cells at various DCs under 0.5 W/cm² Ispta. (D) The uptake of Ru-Lip by total cancer cells at different Ispta levels under 22% DC. Higher apoptotic cell death efficacy with LIPUS application 24 h post-drug administration compared to 20 min in each (E) 5%, (F) 22%, and (G) 45% DC conditions. (H) Comparison of Ru-Lip uptake by cancer cells under 0.5 W/cm² and 0.7 W/cm² Ispta at 45% DC. (I) The number of Gem/Cis-induced apoptotic cancer cells at distances of over 50 μm, 90 μm, and 140 μm from blood vessels based on 0.5 W/cm² Ispta with various DCs. (J) The number of Gem/Cis-induced apoptotic cancer cells at distances of over 50 μm, 90 μm, and 140 μm from blood vessels based on 22% DC with various Ispta levels. (K) The number of cancer cells taken up by Ru-Lip at distances of over 50 μm, 90 μm, and 140 μm from blood vessels based on 0.5 W/cm² Ispta with various DCs. (L) The number of cancer cells taken up by Ru-Lip at distances of over 50 μm, 90 μm, and 140 μm from blood vessels based on 22% DC with various Ispta levels. (M) A 3D plot of Ispta, DC, and the number of Gem/Cis-induced apoptotic cell death (> 50 μm). (N) A 3D plot of Ispta, DC, and the number of cancer cells taken up by Ru-Lip (> 50 μm). Data are shown as mean ± SD, n = 3. * p < 0.05, ** p < 0.01, *** p < 0.001, **** p < 0.0001.

24 h after Gem/Cis administration followed by LIPUS, the total apoptotic cancer cells increased by 2.13-fold, 4.15-fold, and 5.53-fold at DC of 5%, 22%, and 45%, respectively, compared to the control group under the Ispta of 0.5 W/cm^2^ (Figure 8A). Similarly, 24 h after Gem/Cis administration followed by LIPUS, the total apoptotic cancer cells increased by 1.58-fold, 2.21-fold, 4.14-fold, and 4.21-fold at Ispta of 0.1, 0.3, 0.5, and 0.7 W/cm^2^, respectively, compared with the control under a DC of 22% (Figure 8B). Under the Ispta of 0.5 W/cm^2^, the number of total cancer cells taken up Ru increased by 1.75-fold, 4.37-fold, and 5.47-fold at DC of 5%, 22%, and 45%, respectively (Figure 8C). Likewise, at 22% DC, the number of total cancer cells taken up Ru increased by 1.4-fold, 2.42-fold, 4.37-fold, and 4.12-fold at Ispta of 0.1, 0.3, 0.5, and 0.7 W/cm^2^, respectively (Figure 8D).

To determine the best timing for LIPUS irradiation, we compared the efficacy of apoptotic cell death when ultrasound was applied 20 min or 24 h after drug administration. For DCs of 5%, 22%, and 45%, it increased 1.97 times, 3.77 times, and 4.6 times, respectively, at 24 h compared to 20 min (Figure 8E-G). The total number of cancer cells taken up Ru was 43021 and 42975 for Ispta of 0.5 and 0.7 W/cm^2^, respectively, under the 45% DC, showing no significant difference (Figure 8H).

The number of Gem/Cis-induced apoptotic cancer cells at distances over 50 μm with different DCs (5%, 22%, 45%) at 0.5 W/cm^2^ Ispta resulted in increases of 11.3, 12.7, and 18.5-fold, respectively, compared to control (Figure 8I). Additionally, at distances over 90 μm, the number of apoptotic cancer cells were 6, 405, 450, and 1079 at DC of 0%, 5%, 22%, and 45%, respectively. Correspondingly, at distances over 140 μm, the numbers were 0, 16, 25, and 74. The number of Gem/Cis-induced apoptotic cancer cells varying with different Ispta levels (0.1, 0.3, 0.5, 0.7 W/cm^2^) at 22% DC resulted in increases of 6.11, 10, 11.1, and 19.8-fold at distances over 50 μm, compared to control (0 W/cm^2^) (Figure 8J). Additionally, at distances over 90 μm, the numbers were 6, 301, 545, 450, and 1468 at Ispta of 0, 0.1, 0.3, 0.5, and 0.7 W/cm^2^, respectively. Moreover, at distances over 140 μm, the numbers were 0, 27, 101, 25, and 374, respectively. The number of cancer cells taken up Ru with different DCs (5%, 22%, 45%) at 0.5 W/cm^2^ Ispta resulted in increases of 3.67, 10.81, and 15.5-fold at distances over 50 μm, compared to control (Figure 8K). Additionally, at distances over 90 μm, the numbers were 24, 350, 556, and 1093 at DC of 0%, 5%, 22%, and 45%, respectively. Moreover, at distances over 140 μm, the numbers were 0, 23, 107, and 121, respectively. The number of cancer cells taken up Ru with different Ispta levels (0.1, 0.3, 0.5, and 0.7 W/cm^2^) at 22% DC resulted in increases of 3.91, 5.52, 10.81, and 12.85-fold at distances over 50 μm, compared to control (Figure 8L). Additionally, at distances over 90 μm, the numbers were 24, 258, 282, 556, and 868 at Ispta of 0, 0.1, 0.3, 0.5, and 0.7 W/cm^2^, respectively. Moreover, at distances over 140 μm, the numbers were 0, 17, 7, 107, and 26, respectively.

Drug delivery and cell death in the hypoxic region were improved with increasing numbers of DC or Ispta (Figure 8M). The most favorable conditions of Gem/Cis-induced cell death within hypoxia were observed at 0.7 W/cm² Ispta with 22% DC and 0.5 W/cm² Ispta with 45% DC. The delivery of liposomal NPs to the hypoxic region improved with an increase in DC or Ispta (Figure 8N). The most favorable delivery effect of Ru-Lip within hypoxia was observed at 0.5 W/cm² Ispta with 45% DC.

### *In vivo* therapeutic efficacy of LIPUS adjuvant therapy in HuCCT1 CCA

**Figure 9:**
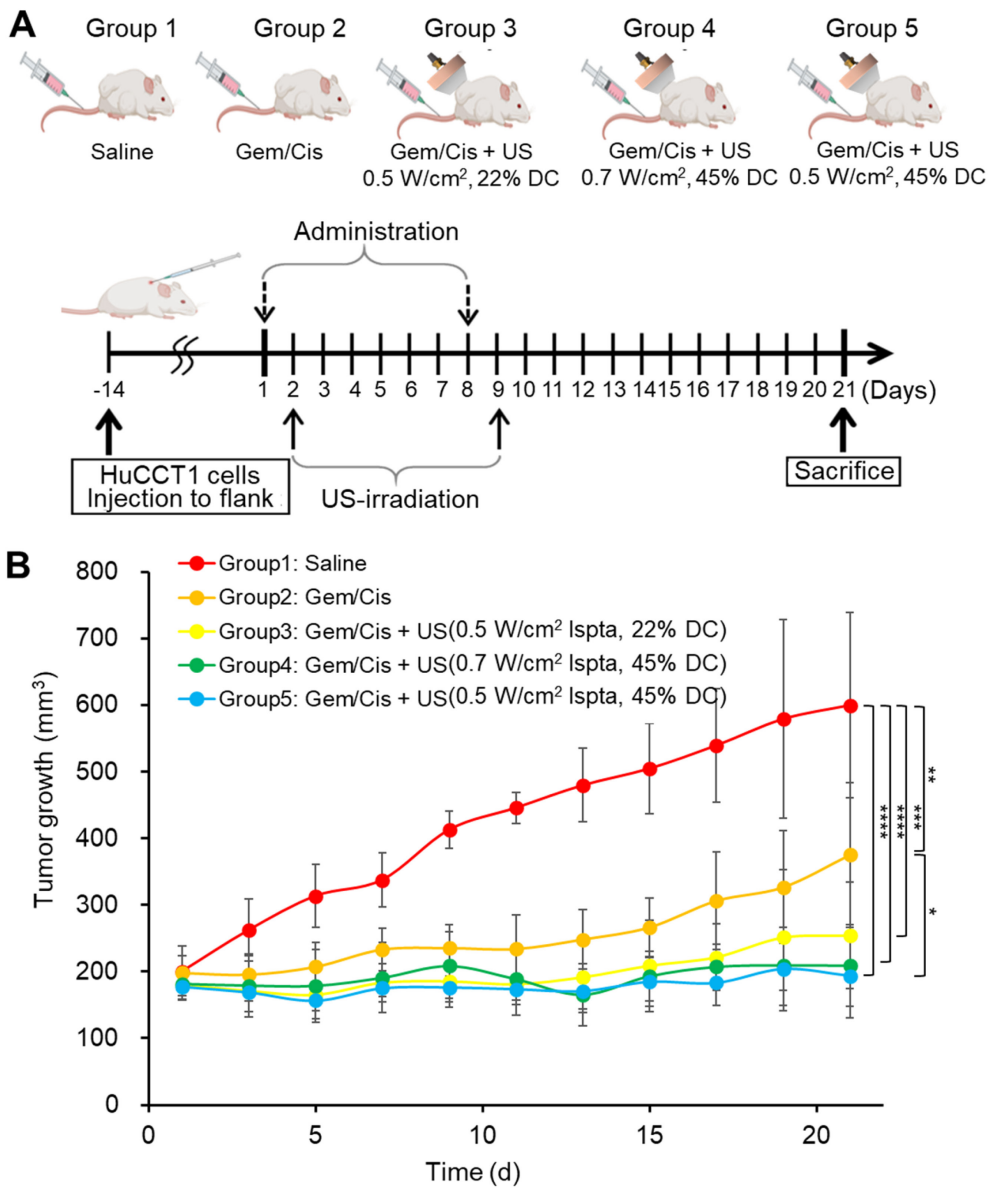
Tumor growth profile assisted by chemotherapeutic agents and LIPUS combination therapy in CCA (HuCCT1). (A) Group 1 is the control group, receiving saline injection into HuCCT1 tumor-bearing mice, while Group 2 received Gem/Cis treatment alone. Groups 3, 4, and 5 were divided into HuCCT1 tumor-bearing mice receiving Gem/Cis treatment concurrently with LIPUS treatment (at 0.5 W/cm² Ispta with 22% DC, 0.7 W/cm² Ispta with 45% DC, and 0.5 W/cm² Ispta with 45% DC, respectively). The mice were administered Gem/Cis on days 1 and 8, and LIPUS was applied 24 h after each drug administration. (B) Tumor growth size was monitored for 21 d every other day.

The antitumor efficacy of the LIPUS-mediated drug treatment was evaluated in the five groups (Figure 9). Compared to group 1 (control), group 2 (Gem/Cis) showed reduced tumor growth owing to the efficacy of the drugs. Especially in groups 3 (0.5 W/cm² Ispta with 22% DC), 4 (0.7 W/cm² Ispta with 45% DC), and 5 (0.5 W/cm² Ispta with 45% DC), wherein LIPUS treatment was synergistically integrated with anti-cancer drugs activity, a controlled modulation of tumor growth rates was evident relative to group 2. Notably, group 5 exhibited the most prominent inhibition of tumor growth, although there was no significant difference between groups 4 and 5.

### Evaluation of LIPUS biosafety and migration effects

**Figure 10:**
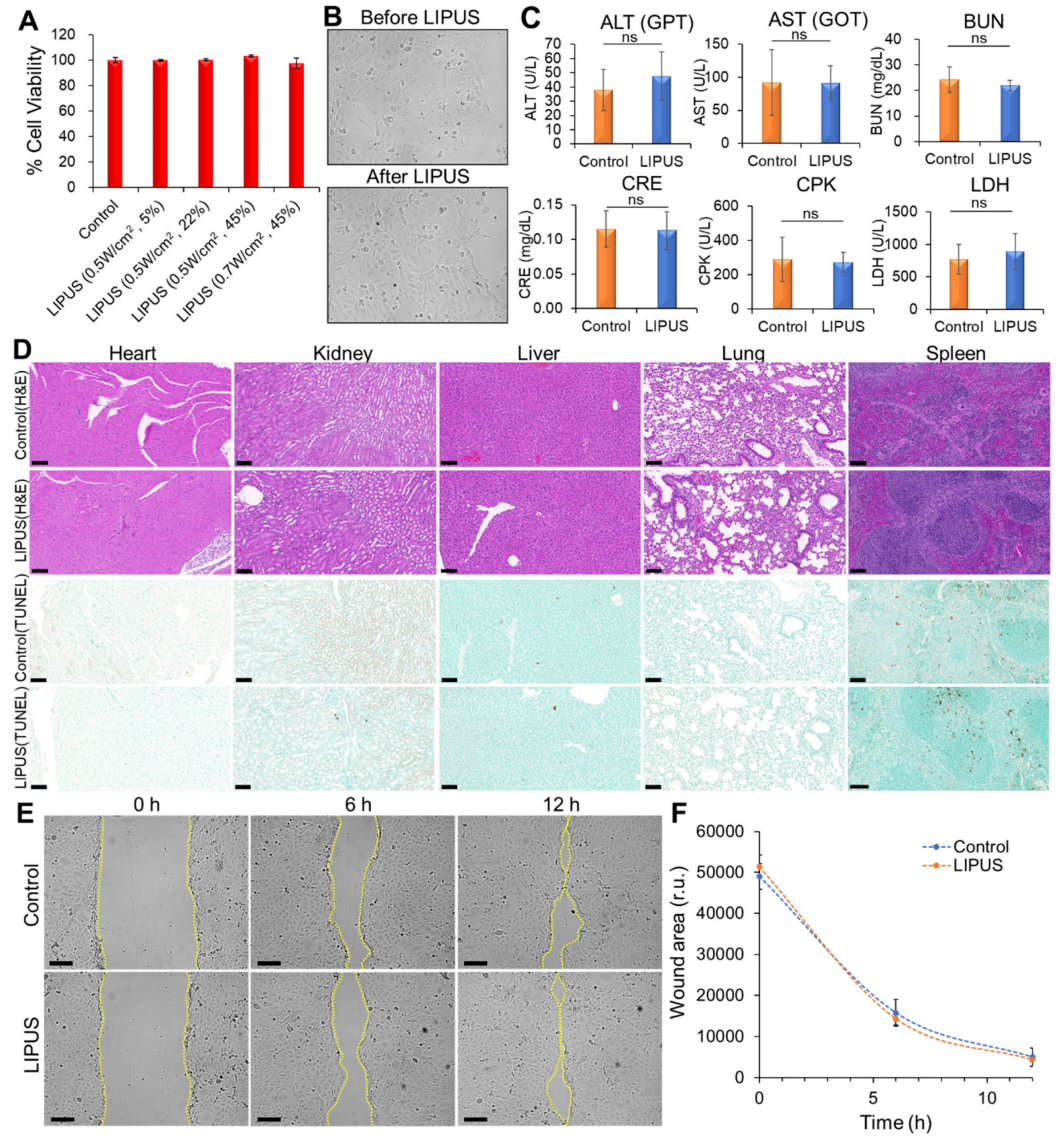
The biosafety of LIPUS *in vitro* and *in vivo*. (A) Cytotoxicity test. HuCCT1 cell viability was assessed by an MTT assay 24 h after LIPUS irradiation. LIPUS conditions: DCs of 5%, 22%, and 45%, Ispta levels of 0.5 W/cm^2^ and 0.7 W/cm^2^. (B) Comparison of cell morphology before and 24 h after LIPUS treatment (0.5 W/cm^2^ Ispta and 45% DC) under brightfield microscopy. (C) The effect of LIPUS treatment (0.5 W/cm^2^ Ispta and 45% DC) on the liver (ALT, AST), kidney (BUN, CRE), cardiac/muscle (CPK), and diverse tissue injuries (LDH). The blood levels of ALT, AST, BUN, CRE, CPK, and LDH in nude mice were measured by biochemical methods. Values are expressed as mean ± SD (n = 4). (D) Representative images of hematoxylin and eosin (H&E) and TUNEL staining of heart, kidney, liver, lung, and spleen tissues from the control group and LIPUS group (0.5 W/cm^2^ Ispta and 45% DC). (E-F) Analysis of HuCCT1 cell migration by wound healing assay. (E) Representative images of wound closure of untreated (top panels) and LIPUS-treated (bottom panels) HuCCT1 cells at 0 h, 6 h, 12 h after cell scratch (LIPUS condition: 0.5 W/cm^2^ Ispta and 45% DC). The dotted lines define the area lacking cells. (F) Quantification of the wounded area invaded by untreated (blue) and LIPUS-treated (orange) HuCCT1 cells over a 12 h period is presented in relative units (r.u.). Values are expressed as mean ± SD (n = 3). All scale bars, 100 μm.

The cytotoxic effects of LIPUS on HuCCT1 cells were evaluated using an MTT assay, which revealed no significant decrease in cell viability across varying DCs (5%, 22%, and 45%) at Ispta levels of 0.5 W/cm^2^ and 0.7 W/cm^2^, indicating that LIPUS treatment did not induce cytotoxicity (Figure 10A). Furthermore, brightfield microscopy showed no noticeable morphological changes in the cells 24 h post-treatment (Figure 10B). Biochemical analysis of serum samples from mice treated with LIPUS (0.5 W/cm² Ispta - 45% DC) showed normal levels of ALT, AST, BUN, CRE, and CPK, indicating no liver, kidney, or cardiac injury (Figure 10C). Additionally, the LDH levels remained stable, suggesting no significant tissue damage. Histopathological examination via hematoxylin and eosin (H&E) staining of major organs (heart, kidney, liver, lung, and spleen) showed no abnormalities in both the control and LIPUS-treated groups, confirming the biosafety of the LIPUS treatment (Figure 10D). TUNEL staining further demonstrated no significant increase in apoptotic cells (Figure 10D). A wound healing assay showed no significant difference in the migration of HuCCT1 cells between the LIPUS-treated and untreated groups over 12 h (Figure 10E-F).

## Discussion

Anti-cancer drugs can rapidly spread throughout the body but have limitations in targeting tumor cells and accumulating at specific sites. Various advanced drug delivery carriers have been developed to overcome these challenges; however, their translation into clinical practice remains limited. CCA is a highly lethal malignancy characterized by significant desmoplastic stroma containing a dense extracellular matrix (ECM) primarily populated by cancer-associated fibroblasts (CAFs) 45. The penetration of drugs and NPs through the dense and stiff ECM in the TME is limited in cancer therapy. Compared with conventional chemotherapy methods or NP delivery systems, the use of therapeutic ultrasound as an external physical stimulus can enhance local drug delivery and improve the efficacy of cancer treatment 46. In this study, LIPUS parameters were optimized to improve the efficacy of drugs or liposomal NPs against CCA. For the first time, we unveiled the efficacy of LIPUS in efficiently delivering anti-cancer drugs or drug-loaded carriers to hypoxic regions within the CCA. Using 3D transparent tissue imaging, the US-assisted distribution of drugs (including liposomal NPs) and apoptotic cell death within the TME were directly visualized and quantitatively analyzed with respect to blood vessels, thereby optimizing the LIPUS parameters to maximize the antitumor effect of LIPUS. The results showed that the actual penetration distance and absorption of drugs varied *in vivo* depending on the DC, acoustic intensity, and treatment time of LIPUS. LIPUS-assisted drug delivery to the CCA resulted in significant tumor suppression.

Presently, there is a growing shift towards utilizing low-intensity ultrasound from high-intensity ultrasound, particularly LIPUS, which is gaining traction from preclinical investigations to actual clinical uses. LIPUS utilizes a non-invasive method of low-intensity pulse-wave stimulation, leveraging its non-thermal effects to deliver safe, cost-effective, and convenient therapeutic benefits. LIPUS increases the permeability of vascular endothelial cells and promotes drug penetration and absorption into tumor tissues through cavitation effects and acoustic streaming 47. Recent studies have demonstrated the potential of low-intensity ultrasound to enhance chemotherapy outcomes, with various approaches being explored. These include combining it with drug-loaded nanoparticles for localized tumor treatment or using microbubble-assisted ultrasonication to improve drug accumulation and efficacy within tumors 15, 48. A study explored the combination of intratumoral injection of drug-loaded magnetic nanoparticles and low-intensity ultrasound using a mathematical framework, showing that it enhances localized chemotherapy and tissue thermal necrosis while minimizing damage to surrounding healthy tissue 15. Additionally, studies have shown that low-intensity ultrasound affects the dissociation of drugs from plasma proteins, thus enhancing the efficacy of chemotherapy 49. Plasma protein binding (PPB) significantly influences drug pharmacokinetics by altering the blood concentration of unbound drugs accessible to target tissues. Previous studies reported non-invasive and spatially-specific disruption of PPB to drugs, using a 250 kHz LIPUS under a 22% DC 41, 42. Cis, the drug used in this study, is associated with protein binding after injection into the bloodstream. In the combination therapy of Gem and Cis used in this study, the plasma half-life of each drug in mice was reported as approximately 17 min for Gem and between 9 to 30 min for Cis 50, 51. Thus, we selected the 20 min time point based on the plasma half-lives of both drugs. It was expected that LIPUS would unbind the drug from plasma proteins and increase drug penetration into the tumor tissue via a gap between tumor vascular endothelial cells. Additionally, LIPUS was applied 24 h after drug injection because within this period, chemotherapeutic agents are typically distributed throughout the body and largely (~ 80%) excreted via urine 50, 51. At this point, we aimed to determine whether LIPUS could enhance drug penetration into deeper regions of the TME after initial distribution within the tumor. Consequently, the drug distributed in the tumor tissue was proven to have improved penetration and absorption into deep tissue by ultrasound, resulting in increased apoptotic cancer cell death in the hypoxic TME compared to the control and 20 min groups. The interaction of ultrasound with tissues includes cavitation and the expansion and contraction of microbubbles, which results in microstreaming characterized by the formation of fluid flows 52. This involves creating pores through sonoporation, allowing anticancer agents to easily enter the cells, a process that is well known to have a positive impact on cancer treatment 53. This is the first report to quantitatively demonstrate an improvement in drug penetration distance within the hypoxic TME of CCA at the micrometer level due to enhanced unidirectional fluid flow by LIPUS. The effects of two ultrasound parameters, DC and Ispta, were evaluated for the enhancement of drug penetration and anti-cancer efficacy. The relationship between the DC, Isppa (spatial-peak pulse-average intensity), and Ispta is expressed as Ispta = DC × Isppa. We predicted that as DC decreased at a constant Ispta, Isppa would increase, leading to increased mechanical forces of the ultrasound and promoting the penetration of the drug into the deep TME owing to increased unidirectional power. However, contrary to initial expectations, the drug penetration distance and efficacy were improved more significantly by DC than by Isppa. Adjusting the DC helps manage the duration of ultrasound exposure and rest periods, thereby minimizing the risk of thermal damage while maximizing non-thermal effects. Typical DC values in LIPUS studies range from 5 to 50% 44, 54. We demonstrated the efficacy of the chosen DCs (5%, 22%, and 45%) in enhancing drug delivery into the hypoxic region of the TME. On the other hand, at the same DC, an increase in Ispta (Isppa) was correlated with improved drug penetration, but saturation was observed at 0.5 W/cm^2^ Ispta or higher intensities with 45% DC. This indicated that a mere increase in Ispta does not necessarily enhance drug penetration efficacy. Therefore, while increases in both Ispta and DC are generally correlated with improved drug penetration in the TME, within the ranges of 0.01 to 0.5 W/cm² for Ispta and 5 to 50% for DC, it can be suggested that higher values will generally enhance drug penetration and efficacy in the hypoxic regions of the TME in CCA.

Ultrasound can be categorized into focused and unfocused modalities, each with distinct advantages and applications. In this study, we used an unfocused ultrasound transducer and employed a hydrogel-filled cone-shaped collimator in the experiments to enhance the targeting and delivery of ultrasound energy. Focused ultrasound concentrates energy on a specific target area, allowing for precise treatment of well-defined tissues, which can be particularly useful in neurosurgery and localized tumor ablation 55. However, such precision has limitations, especially in treating irregularly shaped tumors such as CCA. In contrast, unfocused LIPUS provides broader spatial coverage and consistent mechanical effects across a larger area. This characteristic is advantageous for tumors with diffuse margins, ensuring more uniform drug penetration throughout the TME. This approach also minimizes the risk of creating localized hot spots, thus protecting surrounding healthy tissue while promoting mechanical effects. Moreover, unfocused ultrasound systems are generally more accessible and cost-effective than focused system, making them a viable option for broader clinical use. Our results demonstrate that this method improves drug penetration and therapeutic efficacy in the TME of CCA.

A quantitative approach to assess the drug penetration depth into tumors was explored for the hypoxia region in a previous study 56. However, the authors limited the drug penetration analysis to two-dimensional sections of a 3D tumor. This caused an inherent bias that overestimated the distance between the drugs and the nearest vessel, especially at larger distances, due to the presence of out-of-section vessels (z-axis) 56. Such bias can lead to an overestimation of the drug penetration distance. To minimize the effect of such bias, the authors limited the data fitting to regions within 100 μm of blood vessels. However, in this study, we overcame this limitation by using 3D transparent tumor imaging, to measure drug penetration and the hypoxic region in relation to the nearest blood vessel beyond 100 μm. As tumor vasculature forms a complex 3D network, it is crucial to capture the structure for an accurate analysis. The transparent tumor imaging approach overcomes this limitation by accurately capturing the spatial relationships between tumor cells and blood vessels within the intact 3D tumor volume, providing a more precise and unbiased assessment of the drug penetration distance from the nearest blood vessels. It is well known that conventional thin-tissue section techniques (such as immunohistochemistry (IHC) and *in situ* hybridization (ISH)) are traditionally utilized in many animal studies. However, these methods offer limited insight into the spatial distribution of drugs and their relationship with blood vessels within the TME. When sectioning solid tumors, crucial 3D architectural information such as blood vessel networks is lost. Such destruction of the tissue structure makes it difficult to accurately assess the distribution and efficacy of drugs relative to their proximity to blood vessels. Moreover, due to the irregular nature of tumor vasculature and the random selection of tissue sections, it is challenging to determine the precise escape distance of drugs from blood vessels. This can potentially lead to inaccurate evaluations of the drug penetration and efficacy. To overcome these limitations, we employed the CLARITY tissue optical clearing technique, which allows for the visualization of large, intact tissue regions in 3D without sectioning and reconstruction 57-59. This method enabled us to obtain high-resolution images of drug distribution, apoptotic cell death, blood vessels, and hypoxic regions within intact solid tumors using confocal fluorescence 3D scanning. Unlike traditional methods such as IHC, ISH, and western blotting, this approach preserves the 3D architecture of the tumor, providing a more straightforward, accurate, and spatially resolved analysis of the TME. Additionally, in this study, by utilizing the unidirectional fluid flow property of LIPUS and optimizing LIPUS parameters, we demonstrated the remarkably enhanced drug penetration and therapeutic efficacy in the hypoxic regions of the TME. Under the optimal LIPUS condition, the best growth inhibition effect *in vivo* was observed in a CCA xenograft mouse model.

CCA has a high recurrence rate and is a chemoresistant type of cancer. Tumor hypoxia in CCA can exacerbate this situation because of the inability to reach distant hypoxic regions and gene expression that promotes tumor metastasis or aggressiveness 60. This, in turn, diminishes apoptotic potential and facilitates tumor progression. Conventional chemotherapy or DDS relies on passive drug targeting, driven by the enhanced permeability and retention (EPR) effect. Many solid tumors, including CCA, often develop irregular, leaky blood vessels due to rapid and abnormal angiogenesis, with fenestrations (gaps between endothelial cells) and poor lymphatic drainage. These features enable the EPR effect, allowing drugs or drug-loaded carriers to accumulate in the tumor. However, the EPR effect often results in suboptimal therapeutic outcomes, as drugs tend to remain in the vasculature or penetrate only minimally into the hypoxic regions of the tumor due to characteristics of the TME, such as high IFP and a dense ECM 59. In our study, we aimed to overcome these limitations by using LIPUS to enhance drug distribution in the TME. When applied 24 h after drug injection, LIPUS not only improved drug uptake into cancer cells but also facilitated deeper penetration into the tumor, particularly in hypoxic regions. In a previous study, passively targeted liposomal NPs with varied sizes were found to penetrate less than 100 μm from the nearest blood vessel within skin cancer 59. In contrast, this study shows that LIPUS irradiation facilitates a greater distribution of passively targeted liposomes, allowing them to penetrate over 200 μm into the hypoxic region of CCA (compared to ~ 110 μm for Ru-Lip alone without LIPUS). Furthermore, under optimal LIPUS conditions (DC of 45%, Ispta of 0.5 W/cm²), LIPUS-assisted chemotherapy resulted in approximately 5.5 times more cancer cell deaths compared to chemotherapy alone. The maximum distribution distance of cancer cell death from blood vessels increased by 175%. These findings highlight the potential of LIPUS to overcome the limitations of conventional drug delivery, particularly in targeting hypoxic tumor regions. By enhancing drug delivery into the TME, LIPUS can boost the efficacy of anti-cancer drugs (including drug-loaded NPs), offering a promising approach for optimizing cancer treatment outcomes. Several recent studies have reported the use of LIPUS for enhancing drug delivery in various tumor models. For example, in neuroblastoma models, LIPUS combined with microbubbles (ultrasound contrast agents) significantly increased vascular permeability, improving the uptake of liposomal doxorubicin without causing damage to blood vessels 61. Additionally, LIPUS-guided gene therapy has been used to transiently enhance tumor perfusion, thereby promoting the delivery of liposomal drugs in neuroblastoma via inducible nitric oxide synthase (iNOS) expression 62. In glioblastoma models, LIPUS in combination with microbubbles successfully opened the blood-brain barrier, significantly increasing the concentrations of liposomal doxorubicin and anti-PD-1 antibodies, which in turn potentiated the anti-tumor immune response 63. These findings collectively demonstrate the broad potential of LIPUS-based techniques to enhance drug delivery and therapeutic efficacy across different tumor types including challenging cases where the EPR effect is limited due to barriers like the blood-brain barrier (BBB), such as neuroblastoma and glioblastoma. LIPUS enhances drug delivery by inducing cavitation within tissues, which can lead to the formation and oscillation of microbubbles, without the need for external microbubbles. The mechanical forces generated during cavitation create temporary pores in the cell membrane. These pores increase membrane permeability, facilitating greater intracellular drug uptake (sonoporation). Through this mechanism, LIPUS serves as an effective non-invasive tool for improving drug delivery into target cells. A study found that low-intensity ultrasound can boost the uptake of bisphosphonate (zoledronic acid) into cancer cells by stimulating clathrin-mediated endocytosis 19. This research highlighted that no external microbubbles were involved, nor was the drug encapsulated in liposomes. Instead, LIPUS created mechanical stress and a slight temperature rise, which facilitated the drug's penetration into the cells. Herein, we conducted experiments to investigate the aforementioned mechanisms such as sonoporation and unidirectional fluid flow to support the role of LIPUS in enhancing drug penetration into the hypoxic region of the TME. In one experiment, we demonstrated the unidirectional fluid flow property of LIPUS, showing that LIPUS irradiation enhanced dye infiltration into the deeper regions of the melamine foam (Figure 2D), compared to the setup without LIPUS exposure. This effect is attributed to particle transport driven primarily by LIPUS-induced acoustic streamlines, significantly improving infiltration depth. In contrast, without LIPUS, particle transport was governed by diffusion and thus remained unaffected by acoustic streamlines. Additionally, a separate experiment demonstrated the enhanced uptake of anticancer drugs into cancer cells through sonoporation (Figure 2A-C). The mechanical stimuli also alter cancer cell membrane permeability, thereby increasing drug intake. LIPUS-induced mechanical stimuli increased membrane permeability, which was confirmed by Ru uptake, as observed in confocal imaging and flow cytometry (Figure 2A-C). As a non-invasive and safe adjuvant therapy with no inherent toxicity or thermal effect, LIPUS has the potential to improve patient compliance and treat deep-seated organ tumors, such as the liver, pancreas, and bile duct. Collectively, our results suggest a critical and innovative therapeutic strategy to enhance the low efficacy of chemotherapy or DDS and improve outcomes in patients with cancer, including CCA.

## Conclusion

This study aimed to enhance the efficacy of cancer treatment using highly safe LIPUS to deliver ultrasound waves that penetrate deeply into tissues, thereby increasing the penetration and intracellular delivery of drugs into the hypoxic regions of the CCA. By optimizing LIPUS parameters through transparent tissue imaging, it was found that as DC and Ispta increased, the penetration and efficacy of drugs and carriers increased through enhanced membrane permeability and unidirectional fluid flow. DC had a greater effect on enhancing the drug penetration distance and efficacy than Isppa. Particularly, LIPUS at 45% DC with 0.5 W/cm² Ispta showed the best growth inhibition effect *in vivo* on CCA. Compared to conventional treatments that rely on passive drug targeting driven by the EPR effect, LIPUS offers a clear advantage by enhancing drug distribution, particularly in difficult-to-treat hypoxic tumor regions where conventional therapies fall short. This study demonstrates that LIPUS-assisted chemotherapy can significantly enhance drug penetration and efficacy, resulting in a 5.5-fold increase in cancer cell death compared to chemotherapy alone. Additionally, it facilitates an improved distribution of passively-targeted liposomes. LIPUS has been demonstrated to be a promising and effective strategy for non-invasive cancer treatment, offering significant therapeutic potential for unresectable CCA and other deep-seated organ tumors. As a safe adjuvant therapy with no inherent toxicity or thermal damage, LIPUS can overcome the limitations of conventional drug delivery systems, enhancing the therapeutic efficacy of anti-cancer drugs, including drug-loaded nanoparticles. These findings present LIPUS as a novel and promising therapeutic strategy with the potential to improve outcomes for patients with CCA and other resistant cancers, while also paving the way for broader applications in future cancer treatments.

## Supplementary Material

Supplementary figures and tables.

## Figures and Tables

**Figure 1:**
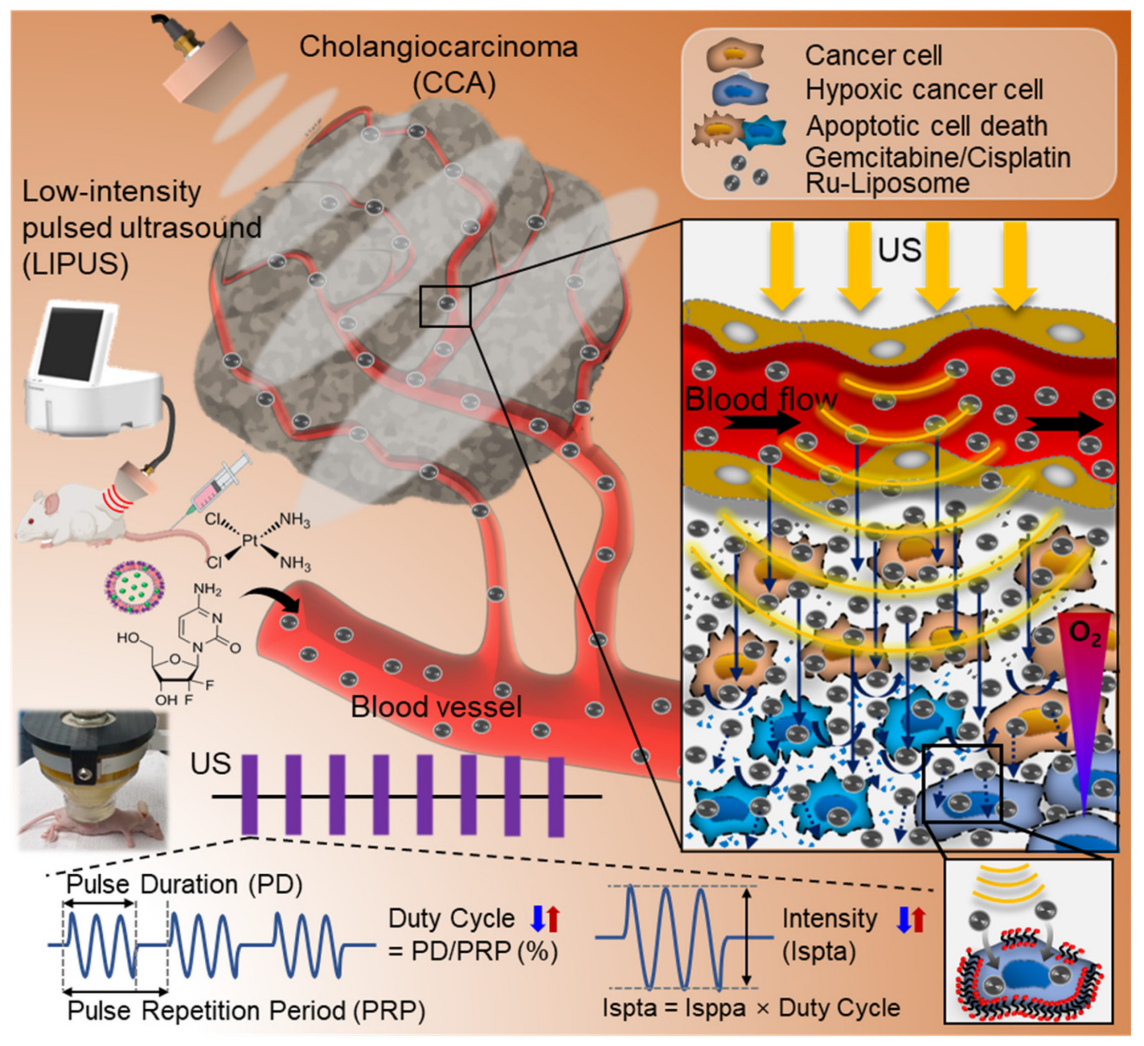
A schematic diagram of the process of administering drugs to mice with cholangiocarcinoma (CCA) and then treating them with low-intensity pulsed ultrasound (LIPUS) under different duty cycle (DC) and spatial-peak temporal-average intensity (Ispta).

## Abbreviations

ALT: alanine transaminase
AST: aspartate transaminase
BUN: blood urea nitrogen
CCA: cholangiocarcinoma
CAF: cancer-associated fibroblast
Cis: cisplatin
CRE: creatinine
CPK: creatine phosphokinase
DDS: drug delivery systems
DC: duty cycle
ECM: extracellular matrix
EPR: enhanced permeability and retention
Gem: gemcitabine
Ispta: spatial-peak temporal-average intensity
IACUC: institutional animal care and use committee
IFP: interstitial fluid pressure
Isppa: spatial-peak pulse-average intensity
LIPUS: low-intensity pulsed ultrasound
LDH: lactate dehydrogenase
NPs: nanoparticles
PFA: paraformaldehyde
Ru-Lip: liposomes-encapsulated ruthenium II
ROI: region of interest
TME: tumor microenvironment
TAM: tumor-associated macrophage
3D: three-dimensional
