# Supplementary material for: Revolutionizing drug delivery: low-intensity pulsed ultrasound (LIPUS)-driven deep penetration into hypoxic tumor microenvironments of cholangiocarcinoma
Source: Theranostics. 2025 Jan 1;15(1):30–51. doi: 10.7150/thno.99981 (PMC11667228; doi:10.7150/thno.99981)
Supplement: Supplementary file 1 — Supplementary figures and tables. [file thnov15p0030s1.pdf]

Supporting Information

Revolutionizing drug delivery: low-intensity pulsed ultrasound (LIPUS)-driven deep penetration into hypoxic tumor microenvironments of cholangiocarcinoma

Sera Hong[1], Jaihwan Kim[2], Gujin Chung[3], Donghyuk Lee[3], and Joon Myong Song[1*]

[1] College of Pharmacy, Seoul National University, Seoul 08826, South Korea

[2] Department of Internal Medicine, Seoul National University Bundang Hospital, Seoul National University College of Medicine, Seongnam 13620, South Korea

[3] Seoul National University Bundang Hospital Healthcare Innovation Park 6F, Seongnam 13605, South Korea

[*] Corresponding author, E-mail: jmsong@snu.ac.kr

Supplementary Figures

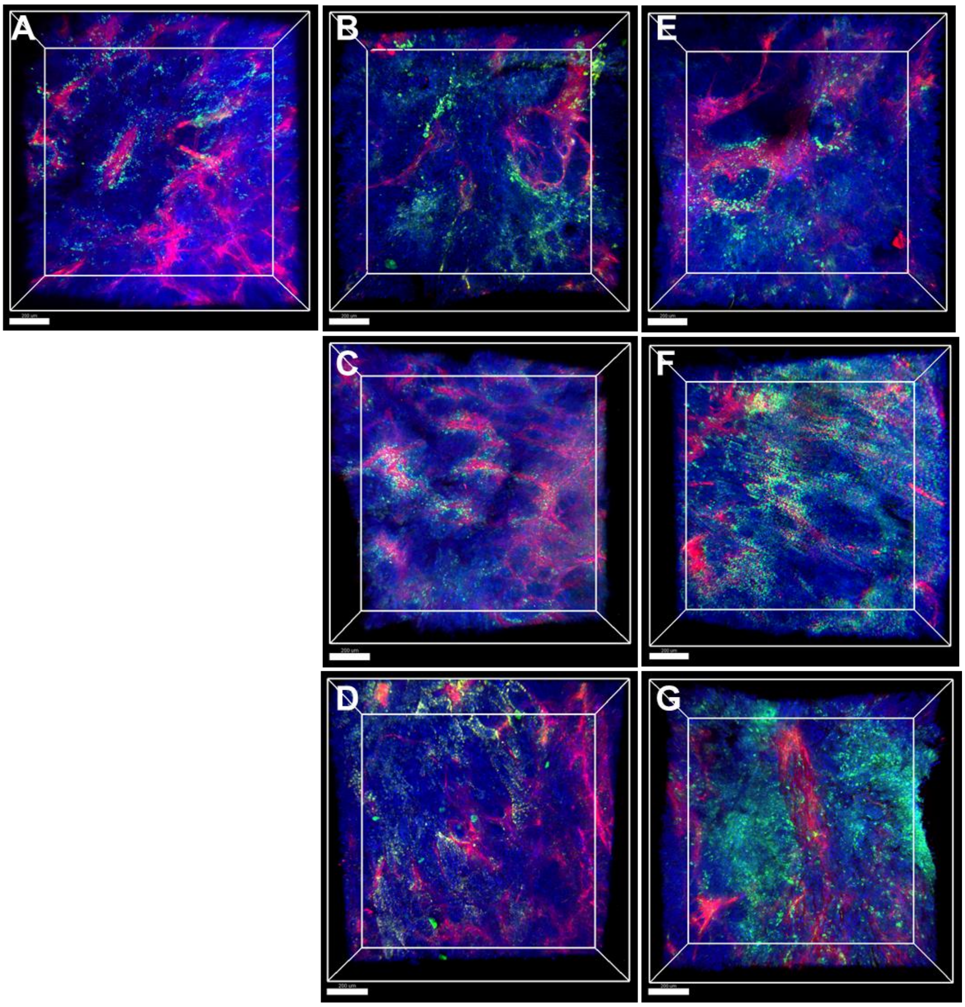

Figure S1. (A) A 3D blood vessel images (red) with TUNEL staining (green) and nucleus (blue) acquired from Gem/Cis-treated HuCCT1 tumor-bearing mice (control) without LIPUS irradiation. (B-G) 3D blood vessel images acquired from mice receiving LIPUS at (B, E) 5%, (C, F) 22%, and (D, G) 45% DCs either (B,C, and D) 20 min or (E, F, and G) 24 h post-drug injection.

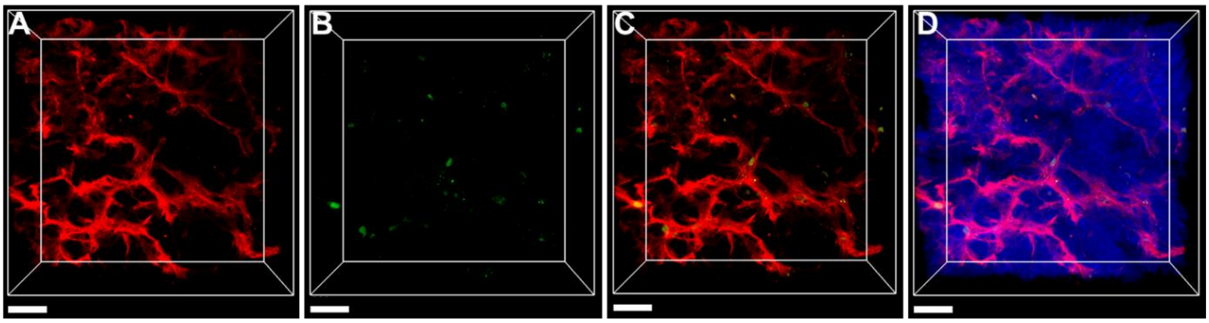

Figure S2. 3D transparent tumor images with TUNEL staining acquired from HuCCT1 tumor-bearing mice receiving LIPUS at 45% DC and 0.5 W/cm^2 Ispta for 1 h without drug injection. 3D distribution of (A) blood vessel (red) alone, (B) TUNEL signal (green) alone, and (C) blood vessel with TUNEL, and (D) blood vessel with TUNEL and nucleus (blue). Scale bars, 200 μm.

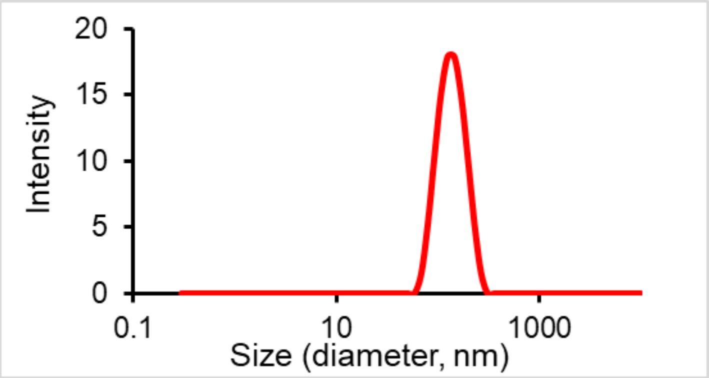

Figure S3. Dynamic light scattering (DLS), Particle size distribution of Ru-Lip.

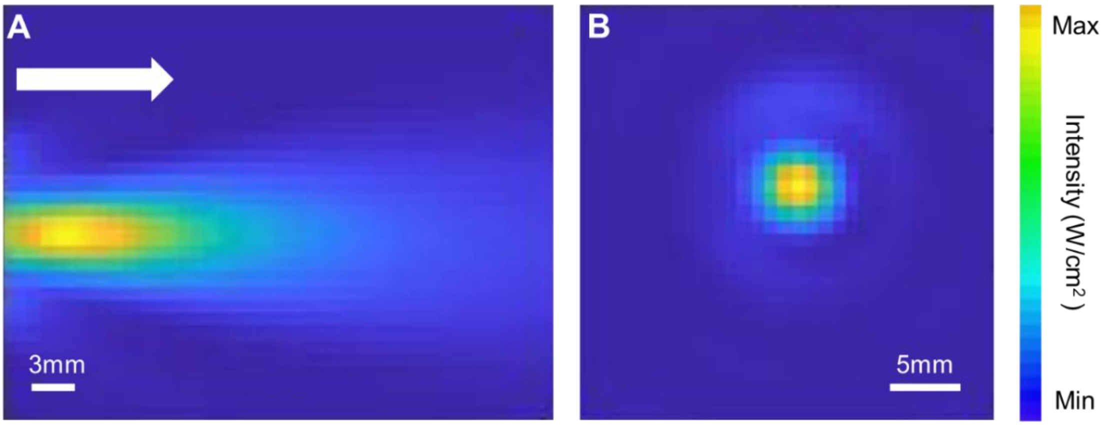

Figure S4. Sonication setup and acoustic intensity profile. The acoustic intensity profile was measured 5 mm away from the surface of the transducer on the (A) YZ axis direction (31 mm ×41 mm; 1 mm step) and (B) XY axis direction (31 mm × 31 mm; 1 mm step). The arrow indicates the direction of ultrasound radiation.

Supplementary Tables

Table S1. LIPUS treatment conditions for animals.

Conditions	Duty cycle (%)	Isppa (W/cm²)	Ispta (W/cm²)	Pascal (kPa)
1	5	9.9	0.5	543
2	22	0.4	0.1	110
3	22	1.4	0.3	203
4	22	2.2	0.5	258
5	22	3.8	0.7	334
6	45	1.1	0.5	180
7	45	1.9	0.7	235

Table S2. Characterization of Ru-Lip.

Sample	Size (nm)	Polydispersity Index (PI)	Zeta Potential (mV)	Encapsulation Efficiency (%)
Ru-Lip	117.6 ± 9.13	0.097	4.519 ± 0.45	90

Table S3. The amount of Gem/Cis and LIPUS combination therapy-induced apoptotic cancer cells in relation to their distance from blood vessels. LIPUS conditions were set dependent on DC (5%, 22%, and 45%) and treatment-time (20 min and 24 h) (Data are presented as mean, n = 3).

Group / Distance from the blood vessels (μm)	Gem/Cis alone	Gem/Cis with US therapy (DC 5%, Ispta 0.5 W/cm^2)		Gem/Cis with US therapy (DC 22%, Ispta 0.5 W/cm^2)		Gem/Cis with US therapy (DC 45%, Ispta 0.5 W/cm^2)	
		20 min	24 h	20 min	24 h	20 min	24 h
10	3345	3038	4366	5180	9387	8766	9893
20	1775	2187	2386	4146	6716	4927	9035
30	560	1339	1760	1588	3978	2228	6202
40	241	867	1313	779	2238	1161	3608
50	128	594	1100	418	1264	624	1936
60	94	448	789	285	820	382	1095
70	69	302	553	176	503	221	774
80	33	183	395	107	352	124	570
90	12	108	265	69	260	65	444
100	3	67	177	44	200	33	381
110	2	49	98	20	119	15	272
120	1	35	53	10	56	12	179
130	0	14	38	7	33	5	113
140	0	10	23	3	16	1	58
150	0	2	11	3	9	2	28
160	0	0	4	0	8	2	17
170	0	0	1	0	6	1	12
180	0	0	0	0	2	0	7
190	0	0	0	0	0	0	7
200	0	0	0	0	0	0	2
210	0	0	0	0	0	0	1
SUM	6263	9243	13334	12835	25967	18569	34637

Table S4. The amount of Gem/Cis and LIPUS combination therapy-induced apoptotic cancer cells in relation to their distance from blood vessels. LIPUS conditions were set dependent on Ispta (0.1 W/cm^2, 0.3 W/cm^2, 0.5 W/cm^2, 0.7 W/cm^2) (Data are presented as mean, n = 3).

Group / Distance from the blood vessels (μm)	Gem/Cis alone	Gem/Cis with US therapy (DC 22%, Ispta 0.1 W/cm^2)	Gem/Cis with US therapy (DC 22%, Ispta 0.3 W/cm^2)	Gem/Cis with US therapy (DC 22%, Ispta 0.5 W/cm^2)	Gem/Cis with US therapy (DC 22%, Istpa 0.7 W/cm^2)
10	3345	3668	5810	9387	9282
20	1775	2235	2386	6716	5901
30	560	1260	1547	3978	3443
40	241	833	1093	2238	2120
50	128	620	868	1264	1398
60	94	393	636	820	1028
70	69	280	436	503	755
80	33	193	312	352	560
90	12	141	212	260	428
100	3	101	154	200	322
110	2	69	97	119	242
120	1	46	71	56	210
130	0	30	66	33	173
140	0	29	56	16	147
150	0	13	43	9	130
160	0	7	23	8	80
170	0	5	19	6	50
180	0	1	8	2	37
190	0	0	5	0	24
200	0	0	0	0	21
210	0	0	0	0	16
220	0	0	0	0	9
SUM	6263	9923	13840	25967	26375

Table S5. The amount of cancer cells taken up by Ru-Lip by LIPUS application in relation to their distance from blood vessels. LIPUS conditions were set dependent on DC (5%, 22%, and 45%) and Ispta (0.5 W/cm^2 and 0.7 W/cm^2) (Data are presented as mean, n = 3).

Group / Distance from the blood vessels (μm)	Ru-Lip alone	Ru-Lip with LIPUS (DC 5%, Ispta 0.5 W/cm^2)	Ru-Lip with LIPUS (DC 22%, Ispta 0.5 W/cm^2)	Ru-Lip with LIPUS (DC 45%, Ispta 0.5 W/cm^2)	Ru-Lip with LIPUS (DC 45%, Ispta 0.7 W/cm^2)
10	4924	7557	11117	12616	9978
20	1388	2244	7164	8854	7889
30	638	1289	5609	7374	7064
40	338	812	3881	5015	5291
50	206	557	2636	3325	4129
60	129	389	1608	2133	2926
70	103	270	991	1318	1920
80	80	206	564	814	1318
90	38	158	324	479	828
100	18	111	185	317	538
110	6	91	109	234	317
120	0	65	74	186	227
130	0	42	45	138	152
140	0	18	37	97	115
150	0	10	34	67	97
160	0	5	24	32	78
170	0	5	17	11	57
180	0	2	15	5	30
190	0	0	10	3	15
200	0	0	5	2	5
SUM	7868	13833	34451	43021	42975

Table S6. The amount of cancer cells taken up by Ru-Lip by LIPUS application in relation to their distance from blood vessels. LIPUS conditions were set dependent on Ispta (0.1 W/cm^2, 0.3 W/cm^2, 0.5 W/cm^2, 0.7 W/cm^2) (Data are presented as mean, n = 3).

Group / Distance from the blood vessels (μm)	Ru-Lip alone	Ru-Lip with US therapy (DC 22%, Ispta 0.1 W/cm^2)	Ru-Lip with US therapy (DC 22%, Istpa 0.3 W/cm^2)	Ru-Lip with US therapy (DC 22%, Istpa 0.5 W/cm^2)	Ru-Lip with US therapy (DC 22%, Ispta 0.7 W/cm^2)
10	4924	4625	7364	11117	12935
20	1388	1941	4010	7164	5529
30	638	1368	2597	5609	4050
40	338	959	1823	3881	2928
50	206	676	1226	2636	2209
60	129	497	759	1608	1574
70	103	327	481	991	1115
80	80	232	318	564	742
90	38	150	226	324	510
100	18	113	135	185	344
110	6	57	81	109	239
120	0	33	39	74	137
130	0	24	15	45	82
140	0	14	5	37	41
150	0	9	4	34	22
160	0	4	2	24	2
170	0	3	0	17	1
180	0	0	1	15	0
190	0	0	0	10	0
200	0	0	0	5	0
SUM	7868	11032	19085	34451	32460
